# Spatial resolution of the metastatic osteosarcoma tumor microenvironment using immunolabeling across murine, canine and human lung

**DOI:** 10.1186/s12967-025-07367-5

**Published:** 2026-02-07

**Authors:** Jessica A. Beck, Janice S. Pereira, Kate I. Silver, Lauren E. McGee, Natalia von Muhlinen, Daniel R. Rissi, Donna O. Butcher, Elijah F. Edmondson, Christina Mazcko, Amy K. LeBlanc

**Affiliations:** 1 Comparative Oncology Program, National Cancer Institute, National Institutes of Health, Building 10, Room 1B53, Bethesda, MD USA; 2 Department of Veterinary Biosciences, The Ohio State University, Columbus, Ohio USA; 3 Laboratory of Human Carcinogenesis, National Cancer Institute, NIH, Bethesda, MD USA; 4 Athens Veterinary Diagnostic Laboratory, Department of Pathology, College of Veterinary Medicine, University of Georgia, Athens, GA USA; 5 Molecular Histopathology Laboratory, Laboratory Animal Science Program, Frederick National Lab for Cancer Research, Frederick, MD USA

**Keywords:** Osteosarcoma, Metastasis, Lung, Animal disease models, Immunohistochemistry

## Abstract

**Background:**

Animal models are crucial resources for studying cancer, metastasis, and the tumor microenvironment (TME). Spatial resolution improves our ability to decipher the functions and interactions of distinct components within the TME. Traditionally, this is accomplished through mapping of protein immunolabeling within tissue sections using immunohistochemistry (IHC). Emerging techniques in this field, including multiplex imaging and spatial transcriptomics, continue to rely on IHC to facilitate cell type and tissue compartment identification. Therefore, appropriate antibody validation remains part of the foundation on which sound and reproducible scientific findings are built. This is important for studies investigating tissue-based hypotheses in murine, canine, or human tissues.

**Methods:**

In this work, we aimed to develop a panel of antibodies to label TME components of metastatic osteosarcoma across murine, canine, and human lung. Candidate antibodies were evaluated across species based on sequence homology, immunolabeling in control samples (positive, negative, isotype), and finally, by western blot.

**Results:**

Herein we present a robust panel of antibodies that label murine, canine, and human immune cells (CD20, CD204, CD3, FOXP3, Iba1), osteosarcoma tumor cells (ALPL, RUNX2, SATB2), and other components of the TME (CD31, cytokeratin, FAPα, PROX1, TTF-1, vimentin), and outline methods in which IHC can be pursued within the context of comparative oncology.

**Conclusion:**

The identification of antibodies that label murine, canine, and human tissues supports the investigation of cancer biology across models and patient populations, highlighting a critical strength of comparative oncology research.

**Supplementary Information:**

The online version contains supplementary material available at 10.1186/s12967-025-07367-5.

## Introduction

Tissues derived from human tumors are a limited resource, making them difficult to study in rare diseases such as pediatric osteosarcoma. Mice are the most frequently used animal model system as they offer several advantages, including a shorter disease time course, ease of genetic manipulation, and the ability to perform controlled drug treatment studies [1, 2]. In parallel, models of naturally occurring disease provide a complementary method to understanding cancer and the co-evolving metastatic tumor microenvironment (TME). The best examples of this approach are canine clinical trials, which offer pet dogs access to cutting-edge research and veterinary care, while simultaneously informing the development of novel therapeutics for humans. These trials provide unique insights into cancer biology and immunology through the study of spontaneous carcinogenesis in an immune-competent host [2, 3]. They also provide critical access to longitudinally collected biologic specimens, including tumor and stroma. Findings from canine clinical trials can then be validated in human tissues and studied experimentally in mouse models. Combining data from all three sources underscores the reproducibility and strength of comparative research.

Our previous work identified an mRNAseq signature in canine osteosarcoma patients that stratifies both canine and human tumors into distinct TME subtypes [4, 5]. These TME subtypes are defined by their stromal (e.g., endothelial cells, fibroblasts) and immune cell (e.g., T cells, B cells, macrophages) composition. Specifically, immune-enriched tumors are associated with improved prognosis [4] except in cases with increased expression of fibroblast and endothelial genes [5], underscoring the complexity of the TME. Critically, these TME subtypes are prognostically relevant in both canine and human osteosarcoma patients and are associated with metastatic progression. To better investigate the spatial distribution of immune cells and stroma, the current study aims to identify antibodies that immunolabel cells and structural components of the osteosarcoma TME across murine, canine, and human lung.

Immunohistochemistry (IHC) is a critical tool for investigating protein immunolabeling within the TME. Immunolabeling can serve as a cornerstone of new studies and corroborate basic science findings in vivo, emphasizing the importance of appropriate antibody validation. However, strategies and methods vary dramatically between publications. Not all scientific journals require IHC controls to be documented or used, which may pose a risk for future studies that expand on scientific literature based on poorly characterized antibodies. It is also a critical concern of the pathology community that exacerbates the well-recognized reproducibility problem in research [6–9]. 

**Fig. 1:**
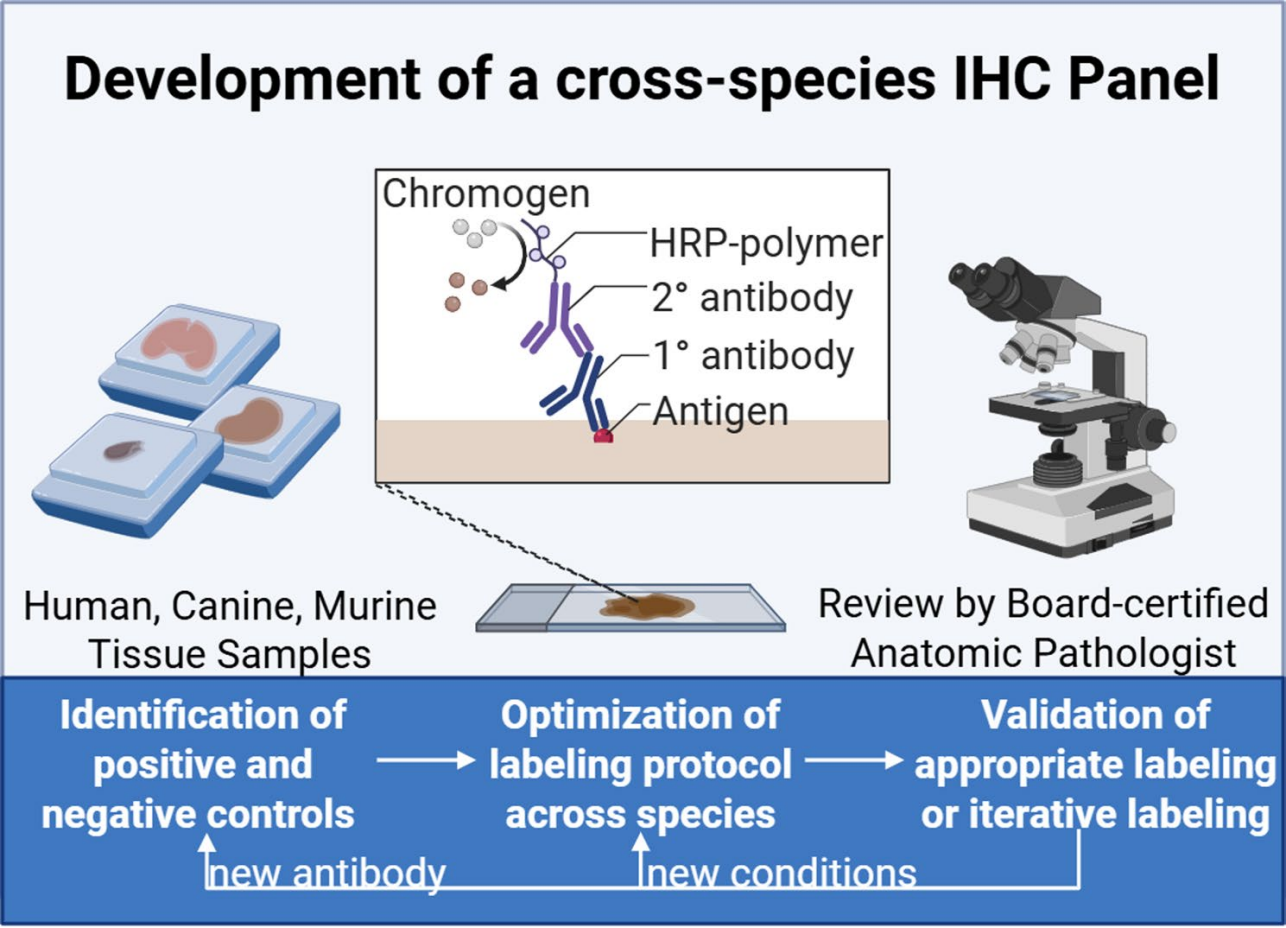
Cross-species Immunohistochemistry. Graphic illustrating the general outline of methods used to test antibodies in murine, canine, and human tissues. After identifying target protein and control tissues, antibodies were selected and tested across murine, canine, and human tissues. Conditions were optimized to determine antibody concentrations, incubation time and temperature, and antigen retrieval methods that yielded appropriate labeling across species. Figure was generated using Biorender

As discussed in a previous review [10], the spatial transcriptomics era has largely focused on mouse and human tissues. Spatial features of interest are often identified through sequential slide hematoxylin and eosin (H&E) staining and IHC. More recent advances have expanded spatial transcriptomics to canine tissues, highlighting the need to improve cross-species antibody validation. Validating techniques and assays across species can be time-intensive and expensive. In the context of IHC, this requires control tissues and optimization of conditions such as antibody concentration and antigen retrieval methods (Fig. 1). However, utilizing reagents solely optimized in a single species can also be cost-prohibitive for comparative oncology studies as it requires a laboratory to maintain assays, methods, and reagent stocks in duplicate or more depending on the number of model species under investigation. Here we identify a panel of antibodies that can be used to label TME targets across murine, canine, and human tissues (Table 1). We evaluated these in lung and included a pneumocyte marker because lung is the most common metastatic site for many cancer types including osteosarcoma. Other tissues and organs of interest could be explored similarly based on our foundational approach.

## Methods

### Sequence homology

Murine, canine, and human protein sequences were obtained from the NCBI Protein Database (https://www.ncbi.nlm.nih.gov/protein) (Supplementary Table 1). When a protein had more than one isoform, the longest isoform sequence was used for the alignment. Protein accession numbers were introduced onto the Multiple Alignment Tool (https://www.ncbi.nlm.nih.gov/tools/cobalt). Protein alignment results were obtained with “Compact” format and “Identity” conversation settings (Supplementary Materials). NCBI Blast (https://blast.ncbi.nlm.nih.gov/Blast.cgi) was used to obtain protein alignment metrics.

### Immunohistochemistry

Immunohistochemical labeling was performed using Leica Biosystems’ BondRX autostainer and Bond Polymer Refine Detection Kit (LeicaBiosystems #DS9800) for each of the antibodies in all three species (murine, canine, and human) at the dilution and antigen retrieval conditions listed in Table 3. Labeling of murine tissues with anti-mouse antibodies was accomplished with the Mouse on Mouse ImmPRESS HRP Polymer Kit (#MP-2400, Vector Laboratories). FOXP3 required manual staining with overnight incubation of the primary antibody. Labeling was examined by a board-certified veterinary pathologist (E.E.) to confirm appropriate intensity, localization, and specificity within a murine, canine, and human control tissue (Table 2; 1 tissue section per species for each marker). Within each tissue, the pathologist reviewed multiple structures. For example, sections of lung contain numerous cells which both express (e.g., type II pneumocytes) or lack (e.g., endothelial cells) TTF-1, allowing for the evaluation of positive and negative labeling within the same tissue section. Isotype controls were used to further test antibody specificity. Antibodies were then applied to FFPE sections containing tumor and non-tumor lung to assess labeling in the lung microenvironment.

### Multiplex imaging

6-plex fluorescent sequential staining of a canine osteosarcoma sample was performed on the Bond RX autostainer (Leica Biosystems) using the Bond Polymer Refine Kit (Leica Biosystems DS9800), with omission of the PostPrimary reagent, DAB and Hematoxylin. After antigen retrieval with EDTA (Bond Epitope Retrieval 2), the section was incubated for 60’ with CD31 (abcam #ab28364, 1:100), followed by the Bond Polymer reagent and OPAL Fluorophore 520 (AKOYA). The CD31 antibody complex was stripped by heating with EDTA (Bond Epitope Retrieval 2). The section was then incubated 30’ with CD204 (TransGenic Inc #KAL-KT022, 1:250), followed by ImmPRESS^®^ HRP-conjugated Horse anti-Mouse (Vector Labs) and OPAL Fluorophore 620. The CD204 antibody complex was stripped by heating with Bond Epitope Retrieval 2. The section was then incubated for 30’ with TTF-1 (abcam #ab227652, 1:100), followed by Bond Polymer reagent and OPAL Fluorophore 690. The TTF-1 antibody complex was stripped by heating with Bond Epitope Retrieval 2. The section was then incubated 60’ with CD3 (Bio-Rad #MCA1477, 1:50), followed by ImmPRESS^®^ HRP-conjugated Goat anti-Rat (Vector Labs) and OPAL Fluorophore 570. The CD3 antibody complex was stripped by heating with Bond Epitope Retrieval 2. The section was then incubated for 30’ with SATB2 (Cell Marque #384R-15, 1:50), followed by Bond Polymer reagent and OPAL Fluorophore 480. The SATB2 antibody complex was stripped by heating with Bond Epitope Retrieval 2. The section was then incubated for 30’ with Cytokeratin WSS (DAKO #Z0622, 1:500), followed by Bond Polymer reagent, TSA-DIG, and OPAL Fluorophore 780. The slide was removed from the autostainer, stained with DAPI and coverslipped with Prolong Gold AntiFade Reagent (Invitrogen). Images were captured using the AKOYA PhenoImager whole slide scanner.

### Western blot

Tissues included in western blot experiments mirrored those used for IHC, including liver (ALPL, CD204, FAPα, PROX1, vimentin), spleen (CD3, CD20, FOXP3, IBA1 MUM1, RUNX2), lung (TTF-1, cytokeratin), kidney (CD31), and brain (SATB2). Western blots were completed in singlicate and included lysates collected from murine, canine, and human tissues (Supplemental Figs. 1–4). To harvest protein from canine and murine tissue samples, Pierce RIPA buffer (#89901, Thermo Scientific™) supplied with cOmplete™ Protease Inhibitor Cocktail (#11836170001, Roche) and PhosSTOP™ (#04906837001, Roche) was added to the tissue and homogenized using TissueRuptor II (#9002755, Qiagen). The protein was separated from cellular debris by centrifugation at 12,000 rpm for 10 min at 4 °C. A BCA assay (#A55864, Thermo Scientific™) was performed to quantify total protein. Human protein lysates were obtained from Novus (NB820-59259, NB820-59231, NB820-59232, NB820-59177, NB820-59239). Equal amounts of denatured protein (40 µg) from murine, canine, and human tissues were loaded into 4–15% or 4–20% precast polyacrylamide protein gels (#4561083/# 4561094, Biorad™) and subjected to electrophoresis under reducing conditions. The gels were transferred to a 0.2 μm PVDF membrane (#LC2002, Invitrogen) and blocked with BSA (#BP1600, Fisher™) for 1 h followed by overnight incubation with primary antibody (1:1000) at 4 °C. Membranes were washed with tris-buffered saline with tween 20 (TBST) prior to incubating with secondary antibodies (1:1000) for 1 h at room temperature (Invitrogen: goat anti-rabbit (31460), goat anti-mouse (31430), and goat anti-rat (31470)). Binding of the HRP-linked secondary antibody was visualized by chemiluminescence using the ChemiDoc Imaging System (Biorad™). β-actin (1:2000, mouse IgG1; #MA1-140, Invitrogen) was used as a loading control. Size approximations for each protein were compiled from commercial supplier datasheets and are included alongside each blot image in Supplemental Figures.

## Results

Antibody specificity is supported by the weight of cumulative evidence; this can be strengthened by incorporating multiple approaches including bioinformatics methods [6]. After identifying TME targets of interest through literature review (Table 1), Basic Local Alignment Search Tool (BLAST) was used to assess sequence homology and predict cross-reactivity of antibodies in nontarget species (Supplemental Materials). As compared to the corresponding human sequence, percent identity of murine and canine protein orthologs ranged from 58% to 100% (Supplemental Table 1).

**Fig. 2:**
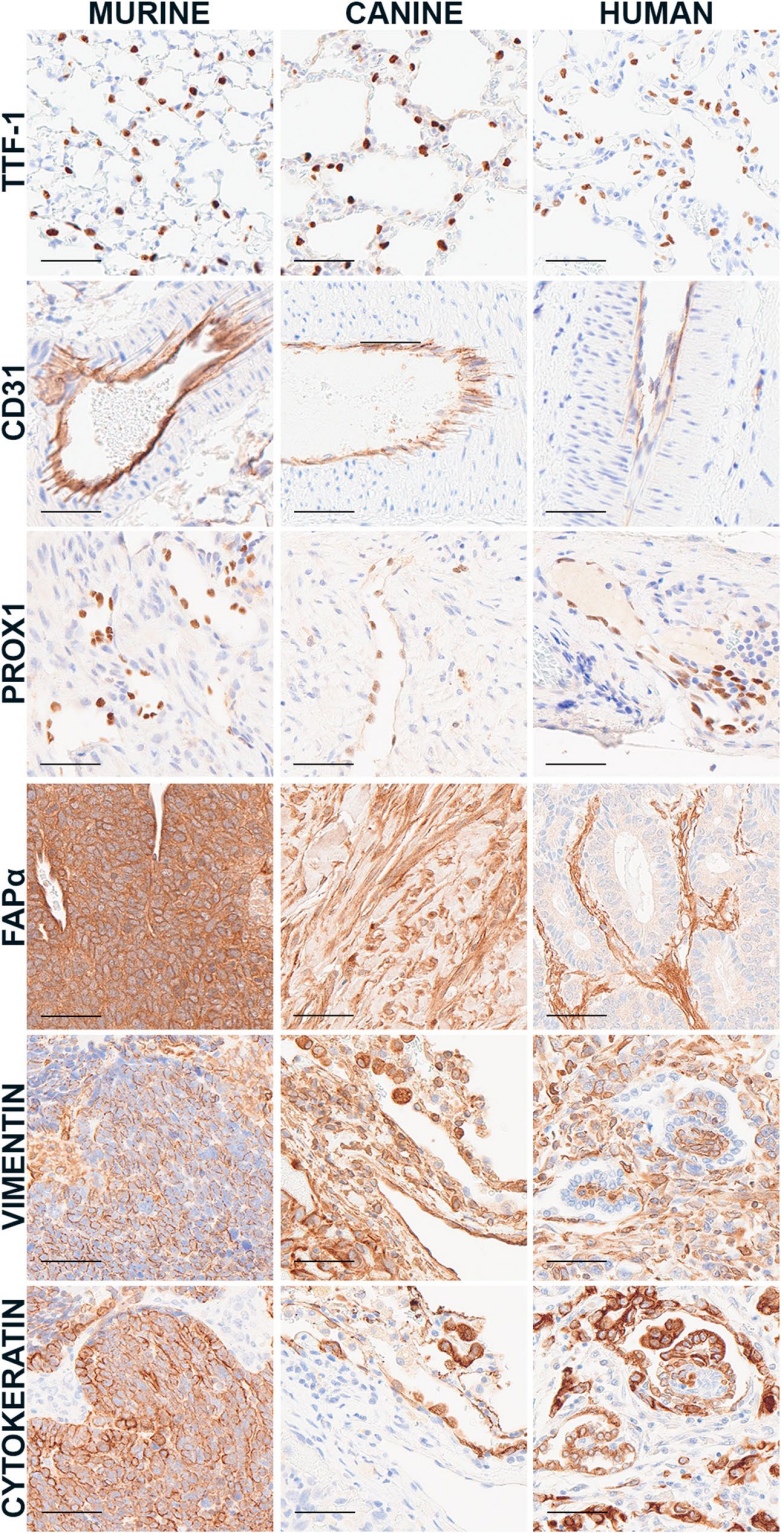
Components of the tumor microenvironment within resident lung. Representative images of immunolabeling of type II pneumocytes (TTF-1; ab227652), endothelial cells (CD31; ab28364), lymphatics (PROX1; 11-002P), fibroblasts (FAPα; ab207178), and mesenchymal (vimentin; ab92547) and epithelial (cytokeratin; Z0622) compartments within murine, canine, and human tissues. Scale bar = 50 μm

We tested our panel of antibodies through IHC labeling of murine, canine, and human control tissues (Table 2). Controls were selected based on the commercial supplier’s datasheet, published literature (Table 1), and the Human Protein Atlas (proteinatlas.org), a useful resource for evaluating expression across multiple human tissues and cell lines [11–13]. When evaluating immunolabeling in control tissues, cells or regions that are negative are as important as those that are positive. For example, nuclear TTF-1 labeling (Fig. 2) was observed in larger epithelial cells within the corners of alveoli but not flattened cells lining alveoli or blood vessels. This finding is consistent with the role of TTF-1 as a transcription factor primarily expressed in type II pneumocytes compared to type I pneumocytes or endothelial cells. For this target, we tested two antibodies (ab227652; M3575); both were labeled across all three species (Fig. 2; Supplemental Fig. 1). We also tested two antibodies directed at CD31 (PECAM1), a transmembrane adhesion molecule with membranous expression in endothelial cells [14]. The first antibody (DAKO M0823) labeled endothelium in human and canine but not murine tissues (Supplemental Fig. 1). Immunolabeling for the second CD31 antibody (Abcam, ab28364) was observed across murine, canine, and human control tissues (Fig. 2). PROX1 was included as a marker for lymphatic endothelial cells [15]; labeling was demonstrated across all three species. Two FAPα antibodies were tested. We were unable to successfully reduce background in two human tissues with the first antibody (Abcam, ab218164); the second FAPα antibody (ab207178) had lower background labeling; however, because this protein is expressed across a variety of cell types and structures (e.g., fibroblasts, granulation tissue, some cancer cells), caution should be taken when interpreting the results (Fig. 2). Finally, vimentin and cytokeratin antibodies were included as general markers of the mesenchymal and epithelial compartments, respectively (Fig. 2).

**Fig. 3:**
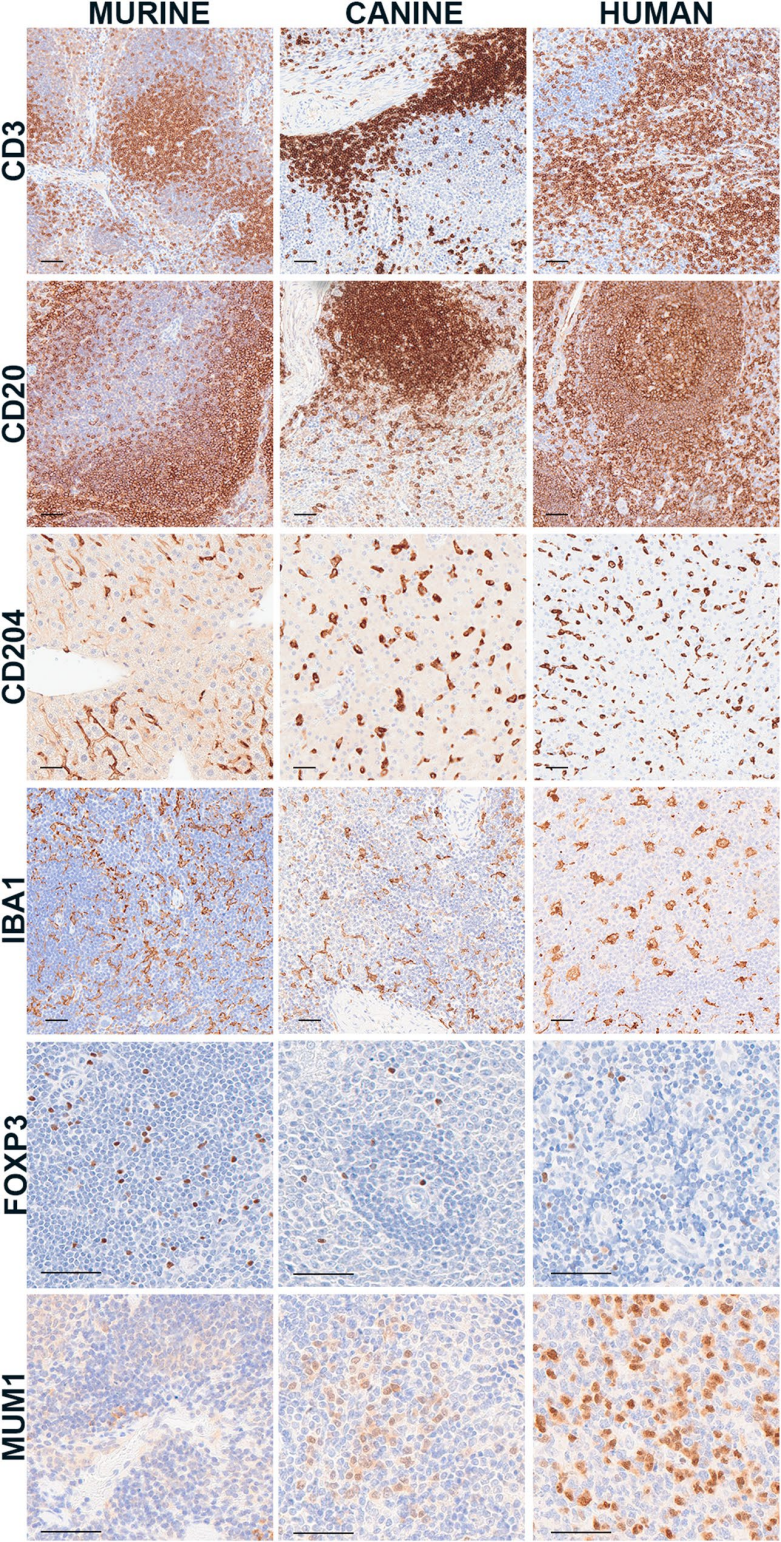
Immune components of the tumor microenvironment. Representative images of immunolabeling of T lymphocytes (CD3; MCA1477), B lymphocytes (CD20; PA5-16701), macrophages (CD204, KAL-KT022; Iba1, CP 290), Tregs (FOXP3; 14-5773), and plasma cells (MUM1; M725929) across murine, canine, and human tissues. Scale bar = 50 μm

We next aimed to identify markers that could be used across species to investigate tumor-directed immunity in the lung TME. The major subsets of immune cells in murine, canine, and human osteosarcoma tissues include T lymphocytes, B lymphocytes, and macrophages; these cells can be labeled across all three species with antibodies targeting CD3, CD20, and CD204, respectively (Fig. 3). CD3 is part of the T cell receptor (TCR)-CD3 complex, which facilitates T cell signaling and activation [16, 17], while CD20 is expressed on pre-B cells and persists until plasma cell differentiation.^34^ Although these antibodies labeled immune cells across all three species, the labeling intensity varied. For example, CD20 was considered sufficiently reactive in human and canine tissues at a lower concentration (0.4 µg/ml); however, this working concentration was increased to improve labeling within the murine positive control. Antibody concentration, incubation, and antigen retrieval methods are included in Table 3. Multiple antibodies were considered for macrophages; CD204 was chosen because it is expressed in tumor-associated macrophages across species and prognostic in several cancer types in human and canine patients [4, 18–20]. We also evaluated Iba1 which has been suggested as a means to stratify M1- and M2-polarized macrophages when used in combination with CD204 [21]. Finally, we tested antibodies directed at two subsets of lymphocytes: T regulatory cells and plasma cells. FOXP3 was reactive across murine, canine, and human tissues. MUM1 labeling was observed in canine and human but not murine tissues.

**Fig. 4:**
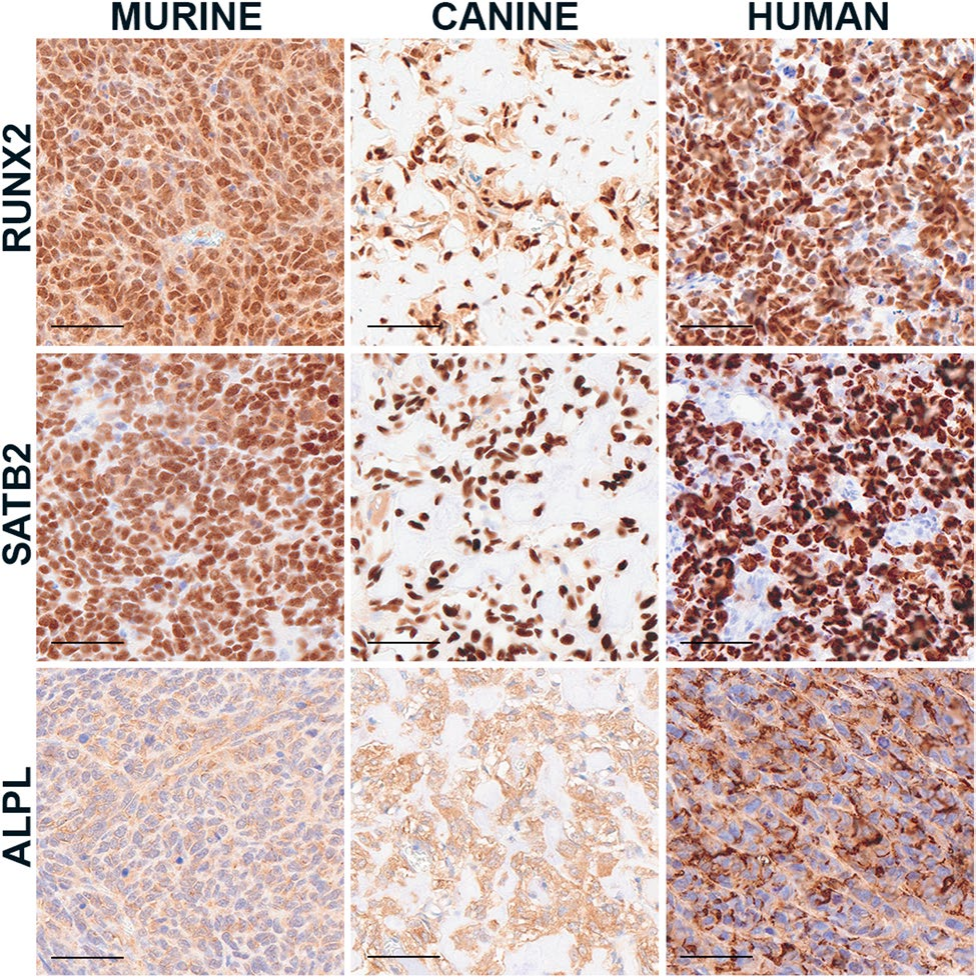
Osteosarcoma tumor markers. Representative images of immunolabeling of RUNX2 (sc-390351), SATB2 (384R-15), and ALPL (ab126820) across murine, canine, and human osteosarcoma cells. Scale bar = 50 μm

**Fig. 5:**
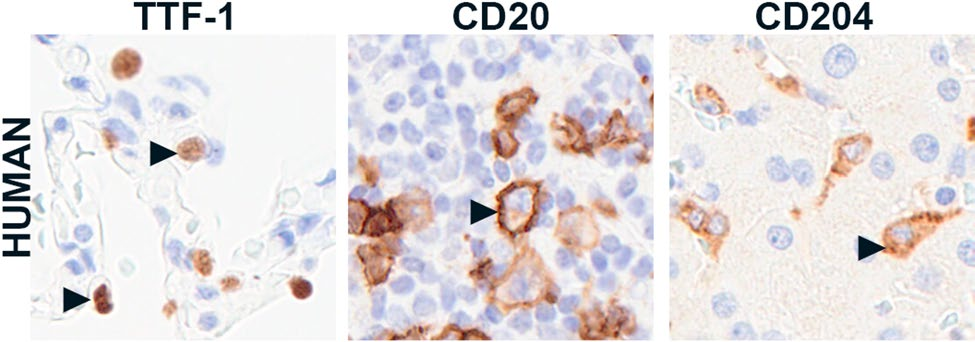
Immunolabeling localization. Representative images of nuclear (TTF-1; ab227652), membranous (CD20; PA5-16701), and cytoplasmic (CD204; KAL-KT022) labeling in human tissues

Because our research focus is osteosarcoma, we also investigated antibodies used to label osteosarcoma cells. Identifying tumor specific protein expression can be challenging because tumor cells share overlapping protein expression profiles with the non-tumor cells from which they are derived. In fact, it is this feature that is often used to identify markers and confirm the lineage of tumors with unusual histomorphology. In the case of osteosarcoma, there are several antibodies targeting osteoblast transcription factors that can be used to label tumor cells across species, including RUNX2 and SATB2 (Fig. 4) [22–26]. Because these are transcription factors, nuclear labeling is observed. This contrasts with ALPL which labels the cytoplasmic membrane of tumor cells (Fig. 4). Nuclear labeling is advantageous as it is focal and distinct from nonspecific background which is often diffuse and cytoplasmic. Nuclear markers are also preferred for spatial analysis because they are easier to segregate and map geographically as compared to cytoplasmic or membranous markers (Fig. 5).

**Fig. 6:**
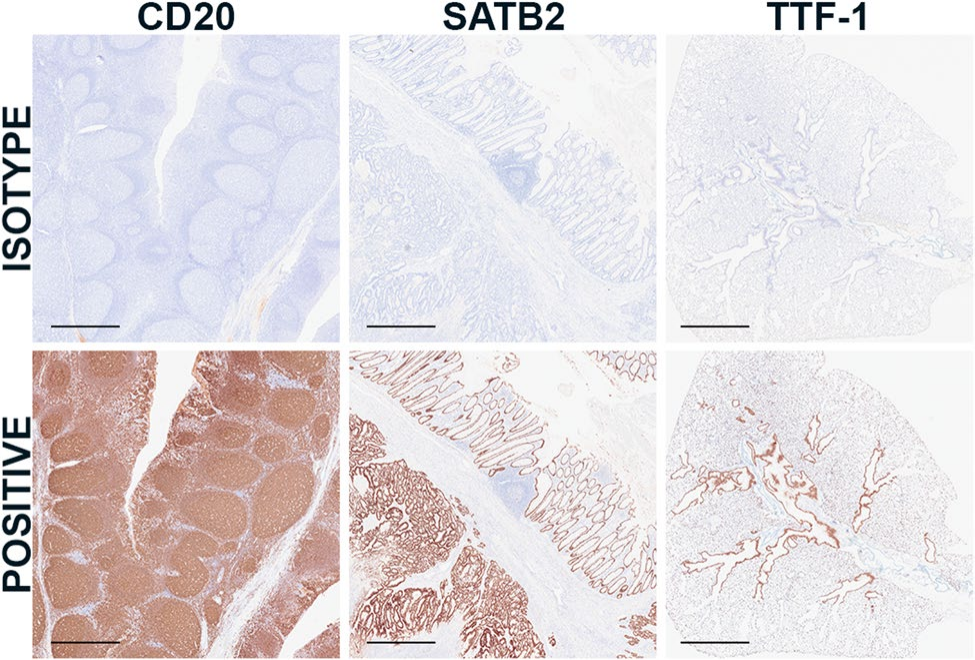
Isotype Controls. Comparison of isotype and positive control labeling for CD20 (PA5-16701), SATB2 (384R-15), and TTF-1 (ab227652) in B lymphocytes (human), colonic epithelium/tumor (human), and lung (murine), respectively. Scale bar = 1.0 mm

In addition to evaluating labeling within positive and negative control tissues, antibody specificity was evaluated using isotype controls. Isotype control labeling was limited across antibodies within our panel; representative images comparing positive and isotype controls are included for CD20, SATB2, and TTF-1 (Fig. 6).

**Fig. 7:**
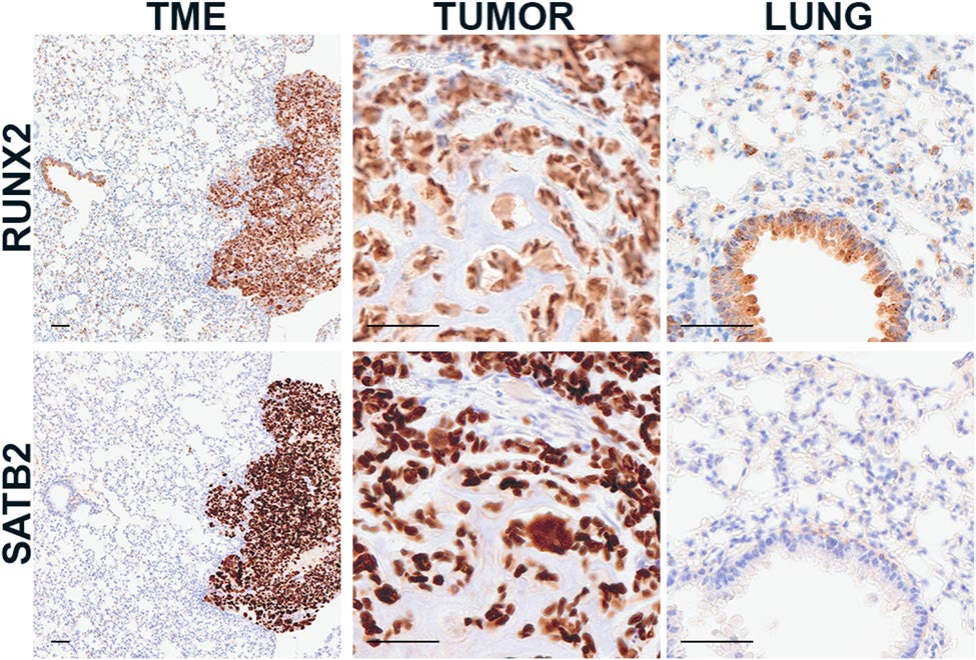
Lung TME. Representative images of RUNX2 immunolabeling (sc-390351) in a metastatic osteosarcoma in murine lung demonstrating nuclear RUNX2 labeling in tumors cells, and in bronchial epithelium and type II pneumocytes in the lung. Nuclear SATB2 labeling (384R-15) in the same tissue occurs in tumor cells but not in type II pneumocytes or bronchial epithelium within the lung. Scale bar = 50 μm

Finally, sections of non-tumor lung were labeled to determine whether any additional lung cells immunolabeled our TME markers. In our panel, RUNX2 was a candidate marker for osteosarcoma cells. RUNX2 is a transcription factor involved in osteoblast differentiation and expressed in the majority of human and canine osteosarcomas [22, 23, 27]; however, our examination of RUNX2 in non-tumor lung also identified expression in bronchial epithelium (Fig. 7). This may be due to the role of RUNX2 in goblet cell differentiation or airway inflammation [28, 29]. Interestingly, RUNX2 was also expressed in type II pneumocytes and is reported to be elevated in type II pneumocytes from patients with pulmonary fibrosis [30]. This highlights the importance of additional non-tumor tissue controls to assess marker specificity within the respective TME. In this case, RUNX2 was found to be less specific for osteosarcoma cells in the lung as compared to SATB2. It is important to note that this will be context specific. For example, SATB2 would be less effective for labeling an intestinal osteosarcoma metastasis due to baseline SATB2 expression in colonic epithelial cells [31].

Here we outline a panel of antibodies optimized for IHC using positive, negative, and isotype controls across three species (murine, canine, and human). In support of our findings, we have included results from BLAST (Supplemental Table 1). All antibodies were also tested by western blot across tissues that mirrored those used in the murine, canine, and human IHC experiments (Supplemental Figs. 1–4). Murine antibodies (ALPL, CD204, CD31, MUM1, RUNX2, TTF-1) demonstrated high background in mouse tissues including bands at 25 and 55 kilodaltons consistent with endogenous IgG. Cytokeratin and vimentin produced bands within the expected range across species (Supplemental Fig. 2); however, variability in the number and size of bands was observed across species for most antibodies. Whole blots are included within the supplemental figures.

**Fig. 8:**
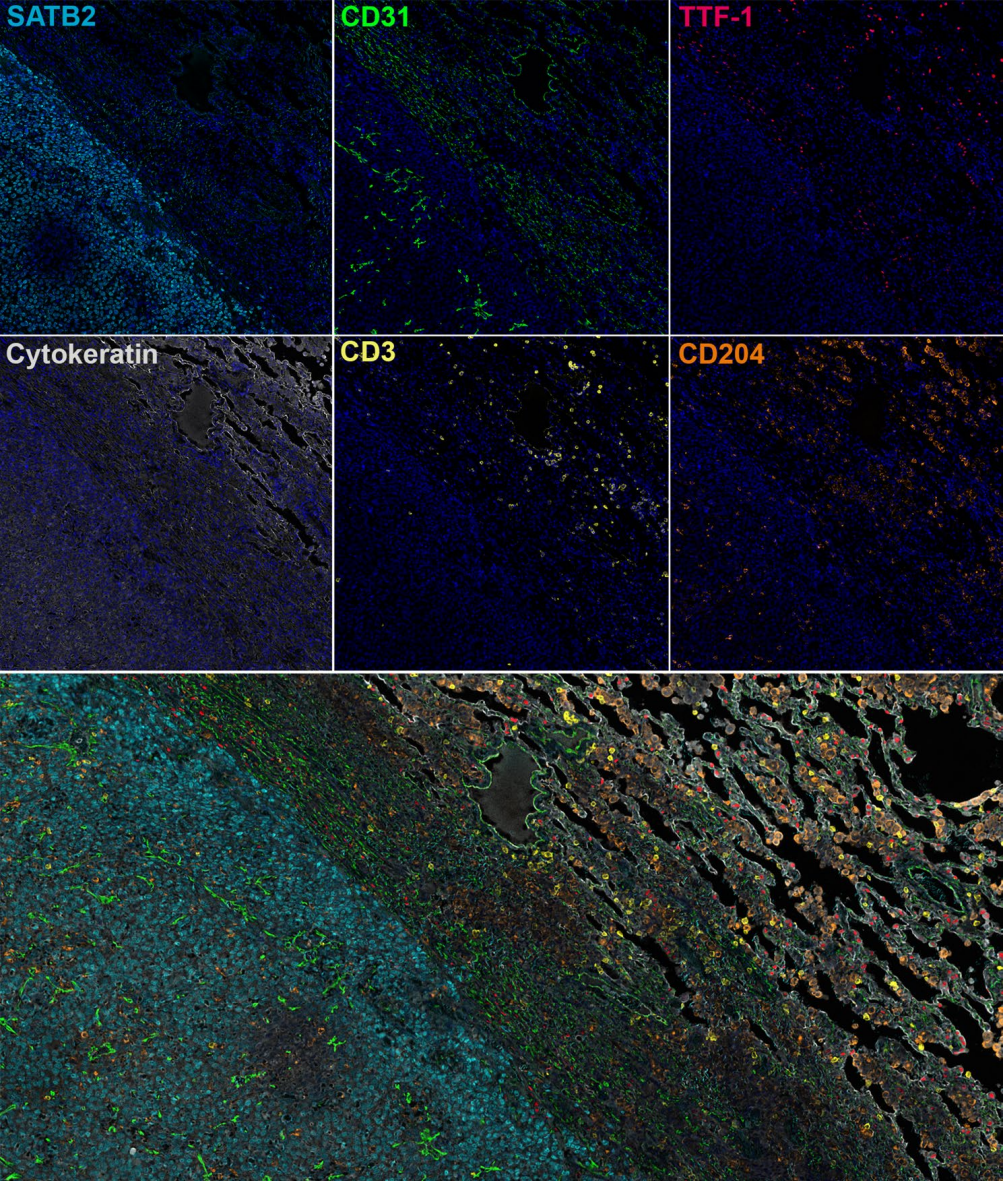
Multiplex Imaging of the canine osteosarcoma TME in the lung. Representative images of metastatic canine osteosarcoma within non-tumor lung labeled for osteosarcoma (SATB2; 384R-15), endothelial cells (CD31; ab28364), type II pneumocytes (TTF-1; ab227652), epithelial cells (cytokeratin; Z0622), T cells (CD3; MCA1477), and macrophages (CD204; KAL-KT022)

Finally, 6-plex fluorescent sequential staining of a canine osteosarcoma sample was performed (CD31, TTF-1, CD3, CD204, SATB2, Cytokeratin; Fig. 8). These images demonstrate the complex spatial architecture of the metastatic osteosarcoma TME in the lung.

## Discussion

Scientific studies routinely incorporate multiple animal models and experimental methods to determine whether a mechanism or target is generalizable or contextual. This often includes cell lines or mouse models but can be strengthened by incorporating human samples or spontaneous models such as the tumor-bearing pet dog. Validation of experimental methods across species improves our ability to pursue these studies by streamlining the workflow and decreasing costs. These efforts also improve visibility of the dog as a naturally occurring cancer patient model which provides unique benefits including an intact immune system and co-evolution of the tumor and TME. This makes them of particular interest to investigators studying anti-tumor immunity or TME-associated therapeutic targets including CAR-T and antibody-drug conjugates [32, 33].

In this study, we identified a panel of antibodies that can be used to label TME components within murine, canine, and human tissues. However, not all antibodies worked similarly across species (Table 3). For example, reduced CD20 labeling was observed in murine tissues compared to human and canine (Fig. 3; Table 3). It is possible that this represents species differences; however, multiple pre-analytical variables [34, 35], such as increased post-mortem interval (PMI), fixation period, or FFPE age, can also reduce immunoreactivity. The impact of these parameters should be assessed through additional controls. For example, comparing labeling within the same tissue fixed for different periods of time allows researchers to understand how fixation affects antibody reactivity (e.g., Ki67 [36]). A similar control is also important in the context of bone-rich tissues, including osteosarcomas, which typically undergo an additional processing step of demineralization prior to embedding and sectioning [37]. In the present study, markers were evaluated for their ability to label specific cell types of interest which is a binary assessment (i.e., yes vs. no). This is because our aim was to facilitate the identification of T cells rather than the amount of CD3 expressed by T cells; however, if the amount of protein per cell is of interest, the noted differences in labeling intensity would preclude direct comparison of expression between species. In this case, incorporating species-specific control samples would be important. Western blot or flow cytometry should also be considered for comparing protein expression between samples.

Additional validation efforts provide confidence in the specificity of an antibody. For example, although it was not the focus of our study, we have included western blot data in supplemental figures. While the role of western blots in IHC validation can be controversial [38–40], they can provide a useful validation step particularly for antibodies with limited or inconsistent literature support [41]. There are several considerations when interpreting western blot results. For example, the absence of a protein band may be due to poor specificity or differences in an antibody’s ability to detect the epitope between IHC and western blot, such as those dependent on the protein’s tertiary structure. In contrast, the presence of multiple bands may be off-target (homologous proteins or paralogs) or specific (e.g., splice variants or post-translational modifications, protein degradation/cleavage). Protease degradation [42] is an important consideration when encountering multiple bands in postmortem tissues; reduced PMI can improve this. The potential for background or off-target labeling emphasizes the need to test antibodies in relevant tissue types and to pursue methods to reduce background, including the use of mouse-on-mouse protocols where appropriate. Methods for improving western blot analysis include the incorporation of good control samples such as cells overexpressing the protein of interest or cells in which a protein has been reduced through knockdown (e.g., siRNA); these experiments are better positioned to identify specific protein bands when multiple bands are observed. If multiple bands persist after western blot troubleshooting, electroblotted proteins may require further investigation (e.g., mass spectrometry [43, 44]). Finally, it’s important to note that antibody performance may be context dependent with discrepancies reported between western blots and IHC [39, 45–47] underscoring the importance of providing evidence of appropriate antibody activity within the intended application.

In support of comparative oncology efforts, this work identifies a panel of antibodies that can be used to label TME targets across murine, canine, and human FFPE tissues. When used in combination (Fig. 8), these markers provide spatial resolution to the TME. Multiplexed marker analysis also enhances diagnostic precision by affording additional information for each cell type. For example, not all osteosarcomas express both RUNX2 and SATB2 highlighting the value of using multiple tumor markers. In addition, although RUNX2 was tested as an osteosarcoma marker, it also labeled adjacent airway epithelium. If this marker is used in combination with TTF-1, pulmonary epithelium (RUNX2+/TTF1+) and osteosarcoma cells (RUNX2+/TTF1-) could be differentiated based on their labeling profiles. Finally, TME subtypes have been shown to be prognostic in osteosarcoma [4, 5]; ongoing studies aim to further characterize the TME to predict therapeutic response or guide immunotherapy strategies in cancer patients [48–50].

For researchers aiming to use this IHC panel, there are several limitations that should be considered. Two of these limitations are discussed above and include (1) differences in labeling intensity between species, and (2) the limited nature of the included western blot analyses. In addition, it is important to note that immunolabeling was evaluated in a single sample per species (e.g., 1 section of canine lung). This allowed for the evaluation of positive and negative labeling in multiple structures within the same sample; however, investigators should consider testing a sample from their cohort to ensure appropriate labeling, which can be impacted by a variety of factors such as prolonged PMI or formalin fixation. Finally, immunolabeling was evaluated by a board-certified pathologist based on known tissue expression. While this method can be useful for evaluating markers with well-characterized expression patterns (e.g., TTF-1 in type II pneumocytes), this method is less effective for labeling cells or components of the TME that cannot be identified based on histology, lack distinct geographic distribution, or are functionally defined. To this point, efforts are ongoing across veterinary and comparative research groups to improve antibody validation in canine tissues, including the development of antibody panels for identifying specific immune cell subtypes in the dog. These efforts are poised to have a profound impact on canine and comparative oncology research.

The acknowledged shortcomings of mouse models increasingly point to the potential value of other species that spontaneously develop cancer in their lifetime. Pet dogs present a unique advantage as a complementary bridge species that can fill knowledge gaps when moving between mice and humans. The panel and methods presented herein provide a roadmap for cross-species work inclusive of the pet dog as a spontaneous patient animal model of human cancer. Tools such as this will help to improve the inclusion of pet dogs in studies of novel cancer drug targets and companion biomarkers for the benefit of human and canine patients.

**Table 1:** Literature review for lung TME targets of interest

TME Targets of Interest	Antibody Candidates	Referncese		
		Murine	Canine	Human
Endothelial Cells	CD31	^[51]^	^[52]^	^[53]^
T lymphocytes	CD3	^[54]^	^[4, 19, 55, 56]^	^[57]^
	FOXP3	^[54]^	^[4, 19]^	^[58]^
B lymphocytes	CD20	^[59, 60]^	^[4, 55]^	^[57, 61, 62]^
	MUM1	^[63]^	^[4]^	^[64, 65]^
Macrophages	CD204	^[66]^	^[4, 19]^	^[66, 67]^
	Iba1	^[66, 68]^	^[4, 55, 69]^	^[66]^
Osteosarcoma	SATB2	^[24]^	^[26]^	^[25]^
	RUNX2	^[70]^	^[23]^	^[71]^
	ALPL	^[72]^	^[73]^	^[74]^
Fibroblasts	FAPα	^[75]^	^[76, 77]^	^[78]^
Type II pneumocytes	TTF-1	^[79]^	^[80, 81]^	^[82]^
Epithelial cells	Cytokeratin	^[83]^	^[84]^	^[85]^
Mesenchymal cells	Vimentin	^[83]^	^[84]^	^[85]^
Lymphatic endothelium	Prox-1	^[86]^	^[87]^	^[15]^

**Table 2:** Control tissues for immunohistochemistry

Antibody	Catalog	Murine Control Tissue	Canine Control Tissue	Human Control Tissue
**ALPL**	ab126820	Liver	Liver	Liver
**CD20**	PA5-16701	Spleen	Spleen	Tonsil
**CD204**	KAL-KT022	Liver	Liver	Liver
**CD3**	MCA1477	Spleen	Spleen	Tonsil
**CD31**	ab28364	Kidney	Kidney	Tonsil
**CD31**	M0823	*Heart, spleen, liver*	Heart, lung	Tonsil
**Cytokeratin**	Z0622	Ovarian carcinoma	Hemangiosarcoma	Mesothelioma
**FAPα**	ab218164	Not tested	Not tested	*Breast & colon carcinoma*
**FAPα**	ab207178	Ovarian carcinoma	Fibrosarcoma	colon adenocarcinoma
**FOXP3**	14-5773	Spleen	Spleen	Tonsil
**Iba1**	CP 290	Spleen	Spleen	Tonsil
**MUM1**	M725929-2	*Spleen*	Spleen	Tonsil
**PROX1**	11-002P	Ovarian carcinoma	Fibrosarcoma	Tonsil
**RUNX2**	sc-390,351	Spleen	Spleen	Tonsil
**SATB2**	384R-15	Brain	Brain	Colon adenocarcinoma
**TTF-1**	ab227652	Lung	Lung	Lung
**TTF-1**	M3575	Lung	Lung	Lung
**Vimentin**	ab92547	Ovarian carcinoma	Hemangiosarcoma	Mesothelioma

^a^CD31 Heart, spleen, liver,  FAPα Breast & colon carcinoma and MUM1 Spleen Tissues within italicized did not demonstrate appropriate antibody immunolabeling with the indicated antibody

**Table 3:** Immunohistochemistry Methods

Antibody	Supplier	Catalog	Working Concentration	Incubation Time/Temp	Antigen Retrieval
**ALPL**	Abcam	ab126820	5.0 µg/ml	30’ RT	Bond EDTA 20’
**CD20**	Invitrogen	PA5-16701	1.6 µg/ml	60’ RT	Bond Citrate 20’
**CD204**	TransGenic Inc	KAL-KT022	1.0 µg/ml	30’ RT	Bond EDTA 20’
**CD3**	BioRad	MCA1477	10 µg/ml	60’ RT	Bond Citrate 20’
**CD31**	Abcam	ab28364	0.13 µg/ml	60’ RT	Bond EDTA 10’
**CD31** ^**a**^	DAKO/Agilent	M0823	10.0 µg/ml	30’ RT	Bond EDTA 20’
**Cytokeratin**	DAKO/Agilent	Z0622	24.6 µg/ml	30’ RT	Bond Citrate 20’
**FAPα**	Abcam	ab207178	7.24 µg/ml	O/N RT	Citrate Buffer 100 C 10’
**FAPα** ^**b**^	Abcam	ab218164	2.5 & 10 µg/ml	60’ RT	Bond Citrate & EDTA 20’
**FOXP3**	eBioscience	14-5773	20 µg/ml	O/N RT	Citrate Buffer 121 C 30”
**Iba1**	Biocare	CP 290	1:500	30’ RT	Bond Citrate 20’
**MUM1** ^**a**^	DAKO/Agilent	M725929-2	9.48 µg/ml	30’ RT	Bond EDTA 20’
**PROX1**	AngioBio	11-002P	5.0 µg/ml	30’ RT	Bond EDTA 30’
**RUNX2**	Santa Cruz	sc-390,351	2.0 µg/ml	30’ RT	Bond EDTA 20’
**SATB2**	Cell Marque	384R-15	3.1 µg/ml	30’ RT	Bond EDTA 20’
**TTF-1** ^**c**^	Abcam	ab227652	1:100	30’ RT	Bond EDTA 20’
**TTF-1** ^**c**^	DAKO/Agilent	M3575	18.0 µg/ml	30’ RT	Bond EDTA 30’
**Vimentin**	Abcam	ab92547	0.13 µg/ml	30’ RT	Bond EDTA 20’

^a^CD31 (M0823) and MUM1 (M725929) labeled canine and human but not murine tissues. ^b^FAPα (ab218164) had higher background labeling and was replaced with ab207178. ^c^Two TTF-1 antibodies were tested to ensure that the panel included a TTF-1 antibody with concentration information

## Supplementary Information

Below is the link to the electronic supplementary material.

Supplementary Material 1

## Data Availability

Data is available upon reasonable request.
