# Correction: Spatial resolution of the metastatic osteosarcoma tumor microenvironment using immunolabeling across murine, canine and human lung

**DOI:** 10.1186/s12967-026-08205-y

**Published:** 2026-05-20

**Authors:** Jessica A. Beck, Janice S. Pereira, Kate I. Silver, Lauren E. McGee, Natalia von Muhlinen, Daniel R. Rissi, Donna O. Butcher, Elijah F. Edmondson, Christina Mazcko, Amy K. LeBlanc

**Affiliations:** 1 Comparative Oncology Program, National Cancer Institute, National Institutes of Health, Building 10, Room 1B53, Bethesda, MD USA; 2 Department of Veterinary Biosciences, The Ohio State University, Columbus, OH USA; 3 Laboratory of Human Carcinogenesis, National Cancer Institute, NIH, Bethesda, MD USA; 4 Athens Veterinary Diagnostic Laboratory, Department of Pathology, College of Veterinary Medicine, University of Georgia, Athens, GA USA; 5 Molecular Histopathology Laboratory, Laboratory Animal Science Program, Frederick National Lab for Cancer Research, Frederick, MD USA

**Correction to: Journal of Translational Medicine (2025) 24:336**

10.1186/s12967-025-07367-5

In the original version of this article, the given and family names of [Jessica A. Beck, Janice S. Pereira, Kate I. Silver, Lauren E. McGee, Natalia von Muhlinen, Daniel R. Rissi, Donna O. Butcher, Elijah F. Edmondson, Christina Mazcko, Amy K. LeBlanc] were incorrectly structured. This has now been amended.

