# Supplementary material for: Spatial resolution of the metastatic osteosarcoma tumor microenvironment using immunolabeling across murine, canine and human lung
Source: J Transl Med. 2026 Feb 7;24:336. doi: 10.1186/s12967-025-07367-5 (PMC12973681; doi:10.1186/s12967-025-07367-5)
Supplement: Supplementary file 1 — Supplementary Material 1 [file 12967_2025_7367_MOESM1_ESM.pdf]

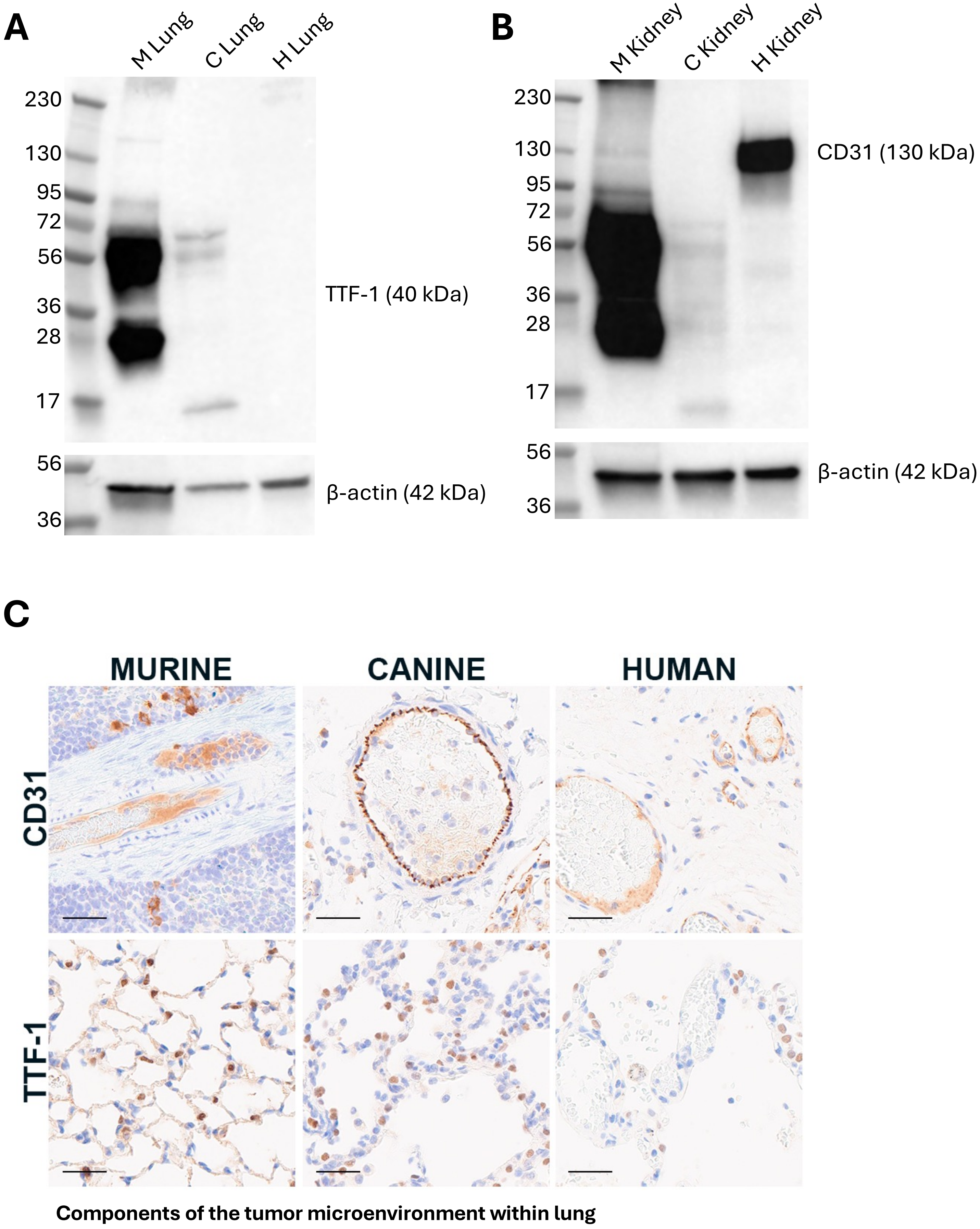

Supplemental Figure 1. Western blots and immunohistochemistry for additional antibodies. Western blot images for (A) TTF-1 (M3575, DAKO/Agilent) and (B) CD31 (M0823, DAKO/Agilent). (C) Representative images of immunolabeling of additional antibodies for type II pneumocytes (TTF-1; DAKO M3575) and endothelial cells (CD31; DAKO M0823).

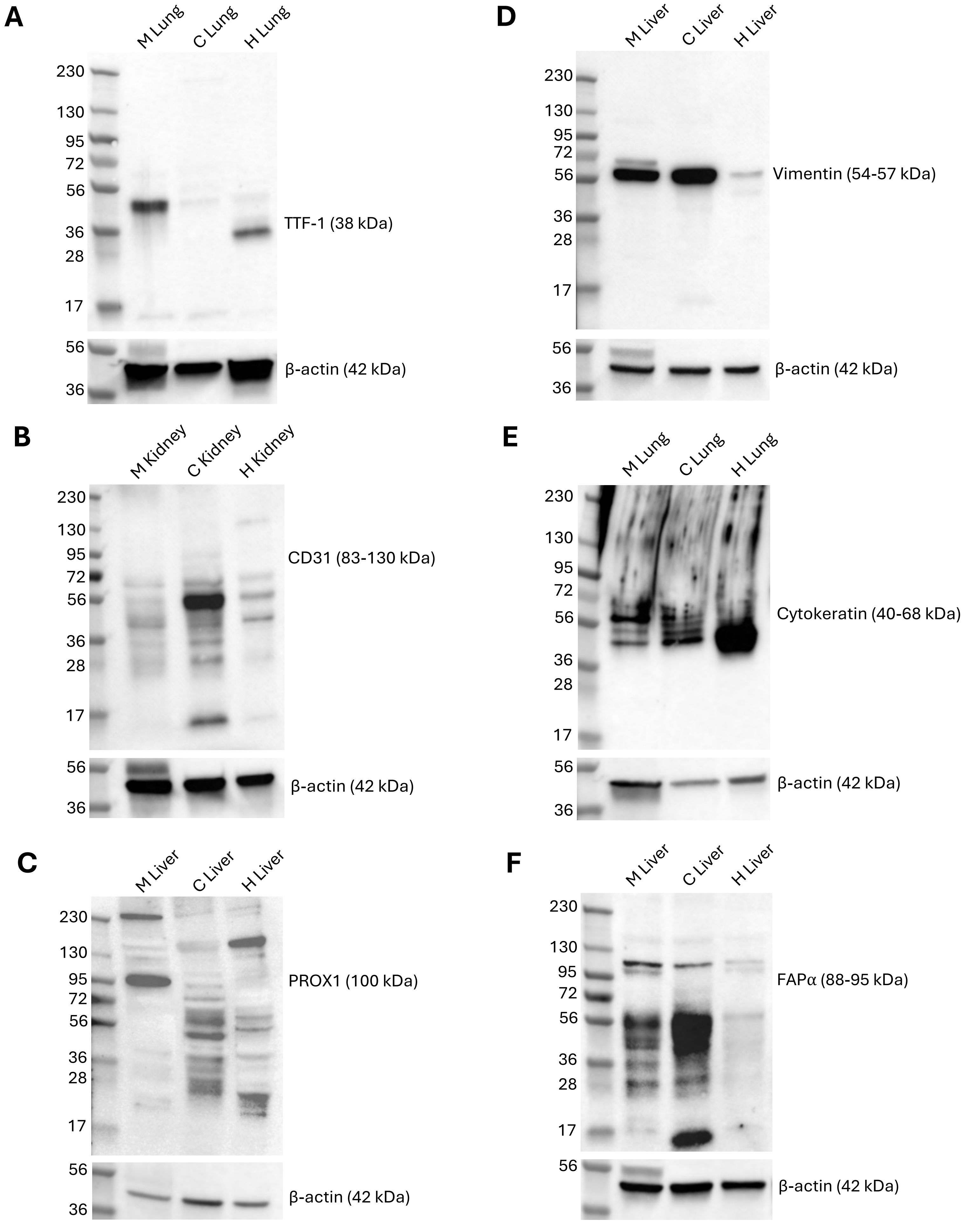

Supplemental Figure 2. Western blots for (A) TTF-1 (ab227652, Abcam), (B) CD31 (ab28364, Abcam), (C) PROX1(11-002P, AngioBio), (D) Vimentin (ab92547, Abcam), (E) Cytokeratin (Z0622, DAKO/Agilent), and (F) FAPα (ab207178, Abcam)

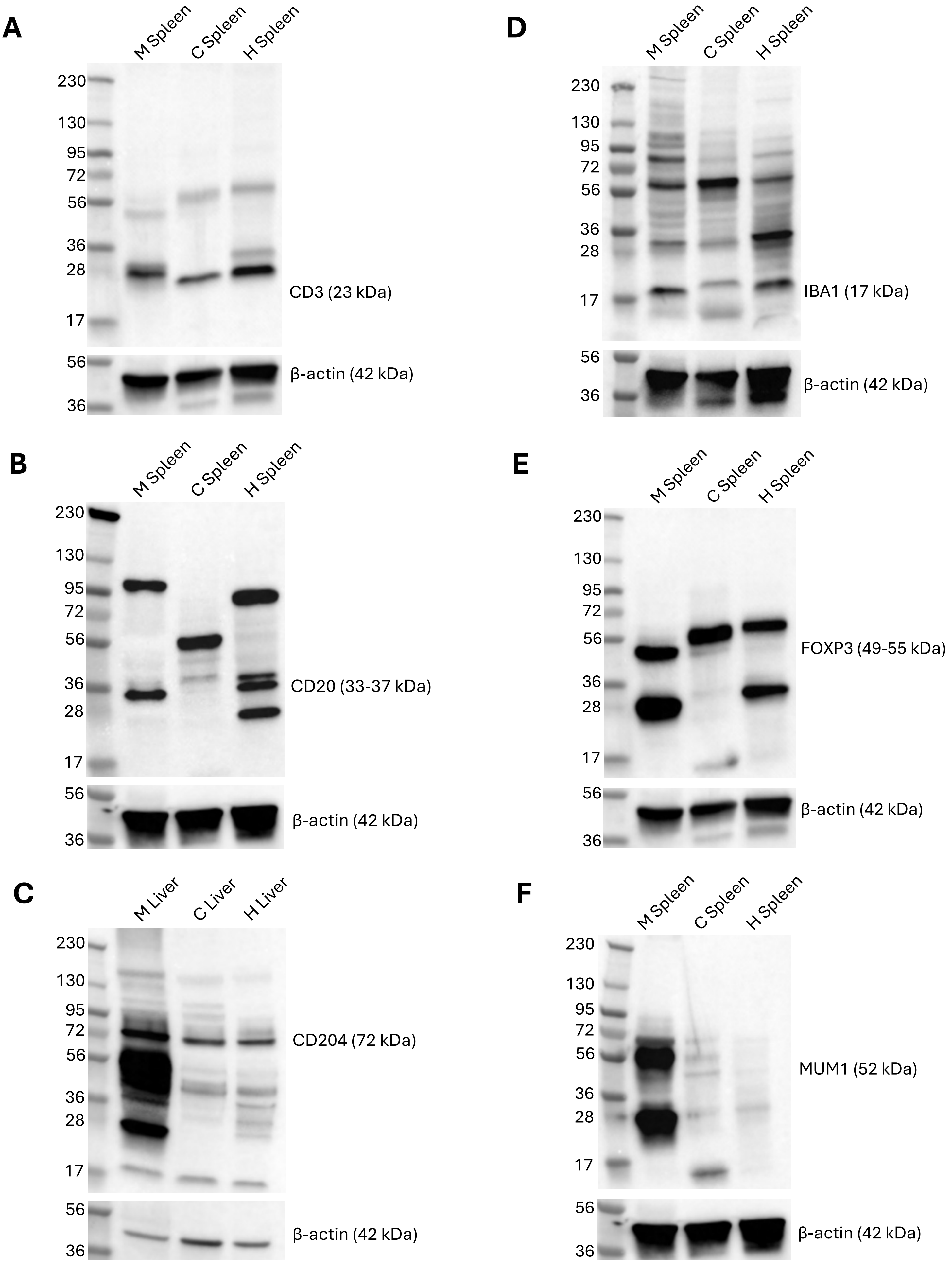

Supplemental Figure 3. Western blots for (A) CD3 (MCA1477, BioRad), (B) CD20 (PA5-16701, Invitrogen), (C) CD204 (KAL-KT022, TransGenic Inc.), (D) IBA1 (CP 290, Biocare), (E) FOXP3 (14-5773, eBioscience), and (F) MUM1 (M725929-2, DAKO/Aligent)

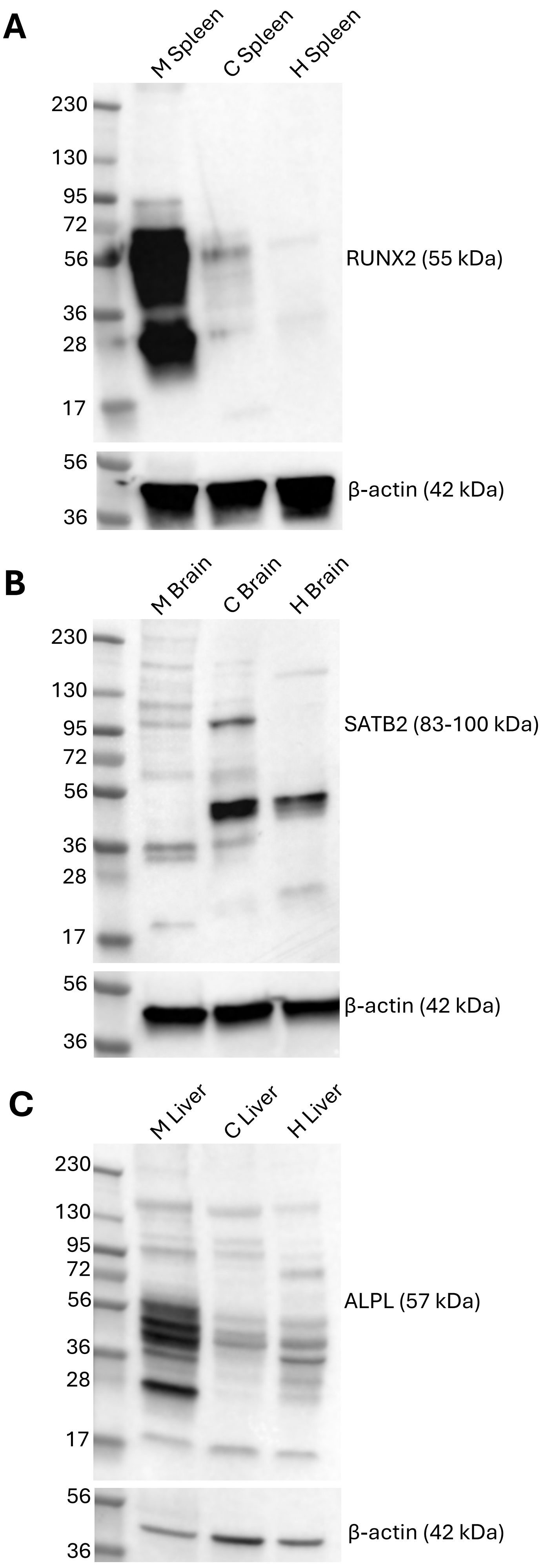

Supplemental Figure 4. Western blots for (A) RUNX2 (sc-390351, Santa Cruz), (B) SATB2 (384R-15, Cell Marque), and (C) ALPL (ab126820, Abcam).

TABLE 1

Description	Scientific Name	Max Score	Total Score	Query Cover	E value	Identity (%)	Acc. Len	Accession	Protein
alkaline phosphatase, tissue-nonspecific isozyme [Canis lupus familiaris]	Canis lupus familiaris	941	941	93%	0	89.47	522	NP_001184066.1	ALPL
alkaline phosphatase, tissue-nonspecific isozyme preproprotein [Mus musculus]	Mus musculus	980	980	97%	0	90.96	524	NP_001416310.1	ALPL
macrophage scavenger receptor types I and II isoform X2 [Canis lupus familiaris]	Canis lupus familiaris	554	554	77%	0	79.94	361	XP_005629987.1	CD204
macrophage scavenger receptor 1 [Mus musculus]	Mus musculus	463	489	74%	3.00E-167	69.55	354	EDL35489.1	CD204
platelet endothelial cell adhesion molecule isoform X1 [Canis lupus familiaris]	Canis lupus familiaris	1066	1066	100%	0	72.26	738	XP_022278549.1	CD31
platelet endothelial cell adhesion molecule isoform 3 precursor [Mus musculus]	Mus musculus	945	945	97%	0	64.39	727	NP_001292086.1	CD31
interferon regulatory factor 4 isoform X8 [Canis lupus familiaris]	Canis lupus familiaris	887	887	100%	0	95.34	451	XP_038319081.1	MUM1
interferon regulatory factor 4 isoform a [Mus musculus]	Mus musculus	882	882	100%	0	92.24	450	NP_038702.1	MUM1
runt-related transcription factor 2 isoform X1 [Canis lupus familiaris]	Canis lupus familiaris	1042	1042	100%	0	98.85	584	XP_038529510.1	RUNX2
runt-related transcription factor 2 isoform 1 [Mus musculus]	Mus musculus	1042	1042	100%	0	97.54	528	NP_033950.2	RUNX2
transcription termination factor 1 isoform X1 [Canis lupus familiaris]	Canis lupus familiaris	789	961	91%	0	71.52	775	XP_005625253.2	TTF-1
transcription termination factor 1 isoform a [Mus musculus]	Mus musculus	683	683	52%	0	71.46	859	NP_033468.2	TTF-1
DNA-binding protein SATB2 isoform X1 [Canis lupus familiaris]	Canis lupus familiaris	109	109	100%	3.00E-34	100	733	XP_005640560.1	SATB2
DNA-binding protein SATB2 isoform 1 [Mus musculus]	Mus musculus	109	109	100%	3.00E-34	100	733	NP_631885.1	SATB2
prospero homeobox protein 1 [Mus musculus]	Mus musculus	108	108	100%	1.00E-33	100	737	NP_032963.1	PROX1
prospero homeobox protein 1 isoform X1 [Canis lupus familiaris]	Canis lupus familiaris	108	108	100%	1.00E-33	100	738	XP_005622418.1	PROX1
vimentin [Canis lupus familiaris]	Canis lupus familiaris	928	928	100%	0	98.07	466	NP_001273952.1	VIMENTIN
vimentin [Mus musculus]	Mus musculus	920	920	100%	0	97.42	466	NP_035831.2	VIMENTIN
prolyl endopeptidase FAP isoform X1 [Canis lupus familiaris]	Canis lupus familiaris	1499	1499	100%	0	93.42	760	XP_005640309.2	FAPα
prolyl endopeptidase FAP [Mus musculus]	Mus musculus	1435	1435	100%	0	89.49	761	NP_032012.1	FAPα
keratin, type I cytoskeletal 20 [Canis lupus familiaris]	Canis lupus familiaris	589	589	100%	0	69.27	434	XP_850091.2	CD20
keratin, type I cytoskeletal 20 [Mus musculus]	Mus musculus	673	673	100%	0	78.42	431	NP_075745.1	CD20
allograft inflammatory factor 1 [Canis lupus familiaris]	Canis lupus familiaris	267	267	100%	2.00E-98	90.48	147	XP_532072.2	IBA1
allograft inflammatory factor 1 isoform a [Mus musculus]	Mus musculus	263	263	100%	1.00E-96	89.12	147	NP_062340.1	IBA1
T-cell surface glycoprotein CD3 epsilon chain isoform X1 [Canis lupus familiaris]	Canis lupus familiaris	244	244	100%	1.00E-87	58.69	202	XP_038364988.1	CD3
T-cell surface glycoprotein CD3 epsilon chain precursor [Mus musculus]	Mus musculus	209	209	100%	7.00E-74	59.42	189	NP_031674.1	CD3
forkhead box protein P3 [Canis lupus familiaris]	Canis lupus familiaris	781	781	100%	0.00E+00	86.12	430	NP_001161933.1	FOXP3
forkhead box P3 [Mus musculus]	Mus musculus	752	752	100%	0.00E+00	82.12	429	AAI32336.1	FOXP3

Mouse and canine protein sequences were aligned to the corresponding human ortholog (query) using NCBI BLAST. Percent of identity (as compared to the corresponding human sequence) is shown.

ALPL

```
NP_001356734.1  1    MISPFLVLAIGTCLTN   SLVPEKEKDPKYWRDQAQETLKYALELQKLNTNVAKNVIMFLGDGMGVSTVTAARILKGQL  77
NP_001416310.1  1    MISPFLVLAIGTCLTN   SFVPEKERDPSYWRQQAQETLKNALKLQKLNTNVAKNVIMFLGDGMGVSTVTAARILKGQL  77
NP_001184066.1  1    ---------MGSCLPP[6]SVYTEKEKDPKYWRDQAQQTLKYALRLQNLNTNVAKNVIMFLGDGMGVSTVTATRILKGQL  74

NP_001356734.1  78   HHNPGEETRLEMDKFPFVALSKTYNTNAQVPDSAGTATAYLCGVKANEGTVGVSAATERSRCNTTQGNEVTSILRWAKDA  157
NP_001416310.1  78   HHNTGEETRLEMDKFPFVALSKTYNTNAQVPDSAGTATAYLCGVKANEGTVGVSAATERTRCNTTQGNEVTSILRWAKDA  157
NP_001184066.1  75   HHNPGEETRLEMDKFPYVALSKTYNTNAQVPDSAGTATAYLCGVKANEGTVGVSAATQRTQCNTTQGNEVTSILRWAKDA  154

NP_001356734.1  158  GKSVGIVTTTRVNHATPSAAYAHSADRDWYSDNEMPPEALSQGCKDIAYQLMHNIRDIDVIMGGGRKYMYPKNKTDVEYE  237
NP_001416310.1  158  GKSVGIVTTTRVNHATPSAAYAHSADRDWYSDNEMPPEALSQGCKDIAYQLMHNIKDIDVIMGGGRKYMYPKNRTDVEYE  237
NP_001184066.1  155  GKSVGIVTTTRVNHATPSAAYAHSADRDWYSDNEMPPEALSQGCKDIAYQLMHNVKDIEVIMGGGRKYMFPKNRTDVEYE  234

NP_001356734.1  238  SDEKARGTRLDGLDLVDTWKSFKPRYKHSHFIWNRTELLTLDPHNVDYLLGLFEPGDMQYELNRNNVTDPSLSEMVVVAI  317
NP_001416310.1  238  LDEKARGTRLDGLDLISIWKSFKPRHKHSHYVWNRTELLALDPSRVDYLLGLFEPGDMQYELNRNNLTDPSLSEMVEVAL  317
NP_001184066.1  235  MDEKSRGTRLDGLNLIDIWKNFKPRHKHSHYVWNRTELLALDPYTVDYLLGLFEPGDMQYELNRNNVTDPSLSEMVEIAI  314

NP_001356734.1  318  QILRKNPKGFFLLVEGGRIDHGHHEGKAKQALHEAVEMDRAIGQAGSLTSSEDTLTVVTADHSHVFTFGGYTPRGNSIFG  397
NP_001416310.1  318  RILTKNLKGFFLLVEGGRIDHGHHEGKAKQALHEAVEMDQAIGKAGAMTSQKDTLTVVTADHSHVFTFGGYTPRGNSIFG  397
NP_001184066.1  315  KILSKNPRGFFLLVEGGRIDHGHHEGKAKQALHEAVEMDRAIGKAGVMTSLEDTLTVVTADHSHVFTFGGYTPRGNSIFG  394

NP_001356734.1  398  LAPMLSDTDKKPFTAILYGNGPGYKVVGGERENVSMVDYAHNNYQAQSAVPLRHETHGGEDVAVFSKGPMAHLLHGVHEQ  477
NP_001416310.1  398  LAPMVSDTDKKPFTAILYGNGPGYKVVDGERENVSMVDYAHNNYQAQSAVPLRHETHGGEDVAVFAKGPMAHLLHGVHEQ  477
NP_001184066.1  395  LAPMVSDTDKKPFTAILYGNGPGYKVVGGERENVSMVDYAHNNYQAQSAVPLRHETHGGEDVAVFAKGPMAHLLHGVHEQ  474

NP_001356734.1  478  NYVPHVMAYAACIGANLGHCAPASSAGSLA-AGPLLLALALYPLSVLF  524
NP_001416310.1  478  NYIPHVMAYASCIGANLDHCAWAGSGSAPS-PGALLLPLAVLSLRTLF  524
NP_001184066.1  475  NYIPHVMAYAACIGANQDHCASASSAGGPSsPGPLLLLLALLPVGILF  522
```

CD204 (MSR1)

```
AAH63878.1      1       MEQWDHF-HNQQEDTDSCSESVKFDARSMTALLPPNPKNSPSLQEKLKSFKAALIALYLLVFAVLIPLIGIVAAQLL  76
EDL35489.1      1    [4]MTENQRLcPHEREDADCSSESVKFDARSMTASLPHSTKNGPSVQEKLKSFKAALIALYLLVFAVLIPVVGIVTAQLL  81
XP_005629987.1  1       MEPWDRF-PDHQDDPDNYSESVKFDARSMTALLPLNPKNGPAVQEKLKSLKAALIILYLLVFVVLIPIIGIMAAQLL  76

AAH63878.1      77   KWETKNCSVSSTNANDITQSLTGKGNDSEEEMRFQEVFMEHMSNMEKRIQHILDMEANLMDTEHFQNFSMTTDQRFNDIL  156
EDL35489.1      82   NWEMKNCLVCSLNTSDTSQGPMEKENTSKVEMRFT-IIMEHMKDMEERIESISNSKADLIDTERFQNFSMATDQRLNDIL  160
XP_005629987.1  77   KWEMKNCTVDPIKANDISQSLTGKGNDSEDEMRFQEVVMEQMSNMEKRIQYISDTEANLIDSEHFQNFSLVTDQRFNDVL  156

AAH63878.1      157  LQLSTLFSSVQGHGNAIDEISKSLISLNTTLLDLQLNIENLNGKIQENTFKQQEEISKLEERVYNVSAEIMAMKEEQVHL  236
EDL35489.1      161  LQLNSLISSVQEHGNSLDAISKSLQSLNMTLLDVQLHTETLNVRVRESTAKQQEDISKLEERVYKVSAEVQSVKEEQAHV  240
XP_005629987.1  157  LQLSTLVSSVRGHGNMIDEISKSLINLNTTLLDLQLNIKTLNDKVQDNTFKQQEDMSRLEERLYNASAEIMSMKEKQVNL  236

AAH63878.1      237  EQEIKGEVKVLNNITNDLRLKDWEHSQTLRNITLIQGPPGPPGEKGDRGPTGESGPRGFPGPIGPPGLKGDRGAIGFPGS  316
EDL35489.1      241  EQEVKQEVRVLNNITNDLRLKDWEHSQTLKNITFIQGPPGPQGEKGDRGLTGQTGPPGAPGIRGIPGVKGDRGQIGFPGG  320
XP_005629987.1  237  EQEIKGEVKLLNNITNDLRLKDWEHSQTLKNITLIQGPPGPPGEKGDRGPTGESGPRGVPGAIGPPGLKGDRGAIGFPGA  316

AAH63878.1      317  RGLP---GYAGRPGNSGPKGQKGEKGSGNTLTPFTKVRLVGGSGPH[92]  451
EDL35489.1      321  RGNPGAPGKPGRSGSPGPKGQKGEKGS------------VGGSRSV      354
XP_005629987.1  317  RGFPGPVGKTGRTGNPGPKGQKGDKGSGSMLRPVQHPDRIW-AGPF      361
```

MUM1 (IRF4)

```
NP_002451.2     1    MNLEGGGRGGEFGMSAVSCGNGKLRQWLIDQIDSGKYPGLVWENEEKSIFRIPWKHAGKQDYNREEDAALFKAWALFKGK  80
NP_038702.1     1    MNLETGSRGSEFGMSAVSCGNGKLRQWLIDQIDSGKYPGLVWENEEKSVFRIPWKHAGKQDYNREEDAALFKAWALFKGK  80
XP_038319081.1  1    MNLEGGGRGGDFGMSAVSCGNGKLRQWLIDQIDSGKYPGLVWENEDKSIFRIPWKHAGKQDYNREEDAALFKAWALFKGK  80

NP_002451.2     81   FREGIDKPDPPTWKTRLRCALNKSNDFEELVERSQLDISDPYKVYRIVPEGAKKGAKQLTLEDPQMSMSHPYTMTTPYPS  160
NP_038702.1     81   FREGIDKPDPPTWKTRLRCALNKSNDFEELVERSQLDISDPYKVYRIVPEGAKKGAKQLTLDDTQMAMGHPYPMTAPYGS  160
XP_038319081.1  81   FREGIDKPDPPTWKTRLRCALNKSNDFEELVERSQLDISDPYKVYRIVPEGAKKGAKQLTLEDPQMTMSHPYTMTAPYTS  160

NP_002451.2     161  LPAQQVHNYMMPPLDRSWRDYVPDQPHPEIPYQCPMTFGPRGHHWQGPACENGCQVTGTFYACAPPESQAPGVPTEPSIR  240
NP_038702.1     161  LPAQQVHNYMMPPHDRSWRDYAPDQSHPEIPYQCPVTFGPRGHHWQGPSCENGCQVTGTFYACAPPESQAPGIPIEPSIR  240
XP_038319081.1  161  LPAQQVHNYMMPPHERGWREYIPDQPHAEIPYQCPVTFGSRGHHWQGPACENGCQVTGTFYACAPPESQAPGIPIEPSIR  240

NP_002451.2     241  SAEALAFSDCRLHICLYYREILVKELTTSSPEGCRISHGHTYDASNLDQVLFPYPEDNGQRKNIEKLLSHLERGVVLWMA  320
```

```
NP_038702.1      241  SAEALALSDCRLHICLYYRDILVKELTTTSPEGCRISHGHTYDVSNLDQVLFPYPDDNGQRKNIEKLLSHLERGLVLWMA  320
XP_038319081.1   241  SAEALALSDCRLHICLYYREILVKELTTSSPEGCRISHGHTYDASNLDQVLFPYPEDNGQRKNIEKLLSHLERGVVLWMA  320

NP_002451.2      321  PDGLYAKRLCQSRIYWDGPLALCNDRPNKLERDQTCKLFDTQQFLSELQAFAHHGRSLPRFQVTLCFGEEFPDPQRQRKL  400
NP_038702.1      321  PDGLYAKRLCQSRIYWDGPLALCSDRPNKLERDQTCKLFDTQQFLSELQVFAHHGRPAPRFQVTLCFGEEFPDPQRQRKL  400
XP_038319081.1   321  PDGLYAKRLCQSRIYWDGPLALCSDRPNKLERDQTCKLFDTQQFLAELQAFAHHGRPLPRFQVTLCFGEEFPDPQRQRKL  400

NP_002451.2      401  ITAHVEPLLARQLYYFAQQNSGHFLRGYDLPEHISNPEDYHRSIRHSSIQE  451
NP_038702.1      401  ITAHVEPLLARQLYYFAQQNTGHFLRGYELPEHVTTP-DYHRSLRHSSIQE  450
XP_038319081.1   401  ITAHVEPLLARQLYYFAQQNSGHFLRGYDLPEHIGSPEDYHRSIRHSSIQE  451
```

RUNX2

```
NP_001019801.3   1        MASNSLFSTVTPCQQNFFWDPSTSRRFSPPSSSLQPGKMSDVSPVVAAQQQQQQQQQQQQQQQQQQQQQQE   A  73
NP_033950.2      1        MASNSLFSAVTPCQQSFFWDPSTSRRFSPPSSSLQPGKMSDVSPVVAAQQQQQQQQQQQQQQQQQQQQQQQ[7]A  80
XP_038529510.1   1    [68]MASNSLFSTVTPCQQNFFWDPSTSRRFSPPSSSLQPGKMSDVSPVVAAQQQQQQQQQQQQQQQQQQQQE----   -  136

NP_001019801.3   74   AAAAAAAAAAAAAAAAAVPRLRPPHDNRTMVEIIADHPAELVRTDSPNFLCSVLPSHWRCNKTLPVAFKVVALGEVPDGTV  153
NP_033950.2      81   AAAAAAAAAAAAAAAAAVPRLRPPHDNRTMVEIIADHPAELVRTDSPNFLCSVLPSHWRCNKTLPVAFKVVALGEVPDGTV  160
XP_038529510.1   137  AAAAAAAAAAAAAAAAAVPRLRPPHDNRTMVEIIADHPAELVRTDSPNFLCSVLPSHWRCNKTLPVAFKVVALGEVPDGTV  216

NP_001019801.3   154  VTVMAGNDENYSAELRNASAVMKNQVARFNDLRFVGRSGRGKSFTLTITVFTNPPQVATYHRAIKVTVDGPREPRRHRQK  233
NP_033950.2      161  VTVMAGNDENYSAELRNASAVMKNQVARFNDLRFVGRSGRGKSFTLTITVFTNPPQVATYHRAIKVTVDGPREPRRHRQK  240
XP_038529510.1   217  VTVMAGNDENYSAELRNASAVMKNQVARFNDLRFVGRSGRGKSFTLTITVFTNPPQVATYHRAIKVTVDGPREPRRHRQK  296

NP_001019801.3   234  LDDSKPSLFSDRLSDLGRIPHPSMRVGVPPQNPRPSLNSAPSPFNPQGQSQITDPRQAQSSPPWSYDQSYPSYLSQMTSP  313
NP_033950.2      241  LDDSKPSLFSDRLSDLGRIPHPSMRVGVPPQNPRPSLNSAPSPFNPQGQSQITDPRQAQSSPPWSYDQSYPSYLSQMTSP  320
XP_038529510.1   297  LDDSKPSLFSDRLSDLGRIPHPSMRVGVPPQNPRPSLNSAPSPFNPQGQSQITDPRQAQSSPPWSYDQSYPSYLSQMTSP  376

NP_001019801.3   314  SIHSTTPLSSTRGTGLPAITDVPRRISDDDTATSDFCLWPSTLSKKSQAGASELGPFSDPRQFPSISSLTESRFSNPRMH  393
NP_033950.2      321  SIHSTTPLSSTRGTGLPAITDVPRRISDDDTATSDFCLWPSSLSKKSQAGASELGPFSDPRQFPSISSLTESRFSNPRMH  400
XP_038529510.1   377  SIHSTTPLSSTRGTGLPAITDVPRRISDDDTATSDFCLWPSTLSKKSQAGASELGPFSDPRQFPSISSLTESRFSNPRMH  456

NP_001019801.3   394  YPATFTYTPPVTSGMSLGMSATTHYHTYLPPPYPGSSQSQSGPFQTSSTPYLYYGTSSGSYQFPMVPGGDRSPSRMLPPC  473
NP_033950.2      401  YPATFTYTPPVTSGMSLGMSATTHYHTYLPPPYPGSSQSQSGPFQTSSTPYLYYGTSSASYQFPMVPGGDRSPSRMVPPC  480
XP_038529510.1   457  YPATFTYTPPVTSGMSLGMSATTHYHTYLPPPYPGSSQSQSGPFQTSSTPYLYYGTSSGSYQFPMVPGGDRSPSRMLPPC  536

NP_001019801.3   474  TTTSNGSTLLNPNLPNQNDGVDADGSHSSSPTVLNSSGRMDESVWRPY  521
NP_033950.2      481  TTTSNGSTLLNPNLPNQNDGVDADGSHSSSPTVLNSSGRMDESVWRPY  528
XP_038529510.1   537  TTTSNGSTLLNPNLPNQNDGVDADGSHSSSPTVLNSSGRMDESVWRPY  584
```

TTF1

```
NP_031370.2      1    MEGESSRFEIHTPVSDKKKKKCSIHKERPQKHSHEI[5]LVNEQSQITRRKKRKKDFQ[4]SPLKKSRICDETANA[5]K  85
NP_033468.2      1    MKGGTSKFKTHTETLYKKKKWSSVSEKRPQKCPSQC   LESKQPQVSVLGKRRRASQ   TPAQETLESEWPQKA   K  71
XP_005625253.2   1    MEGESSRLEIHTPVFD--------------------   -------------------   ---------------   -  16

NP_031370.2      86   RKKRRYSALEVDEEA[6]VDKENINNTPKHFRKDVDVVCVDMSIEQklpRKPKTDKFQVLAKSHAHKSEalHSKVREKKN  168
NP_033468.2      72   RKKRRREPQTPAQET   LESEWPQKAKKKKRRGEPQTPTQESLES------EQPPVSLL--GKRRRES---QTPAQENS  137
XP_005625253.2   17   ---------------   ------------------------------KKKKKKKYSVYKEGHLRHP---HESVRDSPL  44

NP_031370.2      169  KKHQRKAASWE-SQR[6]QSESHQEESWLSVGPGGEITELPASAHKNKSKKKKKKSSNREYETLAMP-------EGSQAG  243
NP_033468.2      138  ESEQPRKAKRRrKKR   KGSQQPTSSLLKTPETFLKAKKTTSAHKKK---KNSVLEVDMETGIILV--DKENMENLLET  209
XP_005625253.2   45   ASEQSRVTKNK-KKR   KDFQHLLSSPLKKSEICDETETATSVPKKKRKKKHRDSGVDEETGVVYVlvDKENIENTPKN  120

NP_031370.2      244  REAGTDM------QESQPTVGlDDETPQL--LGPTHKKKSK------KKKKKKSNHQEFEALAMPEGSQVGSEV-----G  304
NP_033468.2      210  SRKDVDIVYVDMSKGQRSAKV--RETGEL-PAAKPQEHGCREL-LGDVRSRKKQKHLQKVAPWDVVQGSQPESISLPPSE  285
XP_005625253.2   121  FRRDVDVVYVDVSQEQKPAK--DPEAGELhSVPKSHKNELEELnCRVKETKRKKKHRRKEASCDAEQESPPARLALPKSG  198

NP_031370.2      305  ADMQESRP--AVGLHGETAGIPAPAYKNKSKKKKKK-SNHQEFEAVAMPESLESAYP   EGSQVGSEVGTVEGSTALKG  378
NP_033468.2      286  P-------LSSEDLEGKSTEAAVFCKKKS----KKNVFRSQELEPI--PDSLDDSET[5]DSTHHGGAVGAGEECESTK-  353
XP_005625253.2   199  PDPQELEGLLSVGPEGGIPQLPVAADKKKHKKKKRKISHHQELEALAGPGSVENVYS   KGWQRVSEVGTTEEWQGVSE  275

NP_031370.2      379  FKESNSTKKKSKKRKLTSVKRARVS-GDDFSVPSKNSESTLFDSVEGDGAMMEEGVKSRPRQKKTQACLASKHVQEAPRL  457
NP_033468.2      354  --ESHSIKKKSKKKKHKSVALATSS--DSASVTDSKAKNALVDSSEGSGAVREEDVDHRPAEAEAQACSTEKHREAMQRL  429
XP_005625253.2   276  VGTAGRVKKKSKKRKRRSSAESLVMpGGDFTVPATSFEDAHSDSLEGNGALIEESAKPRPQEEKTQACL-----EEVQSL  350
```

```
NP_031370.2      458  EPANEEH-NVETAEDSEIRYLSADSGDADDSDADLGSAVKQLQEFIPNIKDRATSTIKRMYRDDLERFKEFKAQGVAIKF  536
NP_033468.2      430  EPTHEEESNSESASNSAARHISEDRRESDDSDVDLGSAVRQLREFIPDIQERAATTIRRMYRDDLGRFKEFKAQGVAIRF  509
XP_005625253.2   351  EPTNEEEIHLELAKDSETKYLSEDSRDSGDSDVDLDSAVKQLQEFIPDIKERAATTIKRMYRDDLGRFKEFKAQGVAIRF  430

NP_031370.2      537  GKFSVKENKQLEKNVEDFLALTGIESADKLLYTDRYPEEKSVITNLKRRYSFRLHIGRNIARPWKLIYYRAKKMFDVNNY  616
NP_033468.2      510  GKFSAKENKQIEKNVQDFLSLTGIESADKLLYTDRYPEEKTLITNLKRKHAFRLHIGKGIARPWKLVYYRAKKIFDVNNY  589
XP_005625253.2   431  GKFSVKENKQLEKNVQEFLSLTGIENADKLLYTDRYPEEKSVITDLKRKYAFRLHIGKGIARPWKLVYYRAKKMFDINNY  510

NP_031370.2      617  KGRYSEGDTEKLKMYHSLLGNDWKTIGEMVARSSLSVALKFSQISSQRNRGAWSKSETRKLIKAVEEVILKKMSPQELKE  696
NP_033468.2      590  KGRYNEEDTKKLKAYHSLHGNDWKKIGAMVARSSLSVALKFSQIGGTRNQGAWSKAETQRLIKAVEDVILKKMSPQELRE  669
XP_005625253.2   511  KGRYSKGDMEKLKIYHSLHGNDWKKIGEMVARSSLSVALKFSQIGSPRNHGAWSKTETQKLIKAVEEVILKKMSPHELKE  590

NP_031370.2      697  VDSKLQENPESCLSIVREKLYKGISWVEVEAKVQTRNWMQCKSKWTEILTKRMTNGRRIYYGMNALRAKVSLIERLYEIN  776
NP_033468.2      670  LDSKLQEDPEGRLSIVREKLYKGISWVEVEARVETRNWMQCKSKWTEILTKRMTHGGFVYRGVNALQAKITLIERLYELN  749
XP_005625253.2   591  VDSKLQDNPEGCLSIVREKLYKGISWVEVEAKVETRNWMQCKSKWTEILTKRMTNGRDVYRGVNALQAKINLIERLYEVN  670

NP_031370.2      777  VEDTNEIDWEDLASAIGDVPPSYVQTKFSRLKAVYVPFWQKKTFPEIIDYLYETTLPLLKEKLEKMMEKKGTKIQTPAAP  856
NP_033468.2      750  VNDANEIDWEDLASAIGDVPPPFVQAKFYKLKAACVPFWQKKTFPEIIDYLYKNSLPLLKEKLDKKMKKKDGQIQTPAAP  829
XP_005625253.2   671  VEDTNEIDWEDLASAIGDVPPSYVQTKFYKLKATCVPFWQKKTFPEIIDYLYETTLPLLKEKLEKKMEKEGTEIQSPAAP  750

NP_031370.2      857  KQVFPFRDIFYYEDDSEGEDIEKESEGQAP[19]   905
NP_033468.2      830  KQDFLFKDIFHCDDDSDEGSPEEPSASDVQ       859
XP_005625253.2   751  RQVFLFRDIFYRDDDSEGEDGEEKS-----       775
```

SATB2

```
NP_001165980.1   1    MERRSESPCLRDSPDRRSGSPDVKGPPPVKVARLEQNGSPMGARGRPNGAVAKAVGGLMIPVFCVVEQLDGSLEYDNREE  80
NP_631885.1      1    MERRSESPCLRDSPDRRSGSPDVKGPPPVKVARLEQNGSPMGARGRPNGAVAKAVGGLMIPVFCVVEQLDGSLEYDNREE  80
XP_005640560.1   1    MERRSESPCLRDSPDRRSGSPDVKGPPPVKVARLEQNGSPMGARGRPNGAVAKAVGGLMIPVFCVVEQLDGSLEYDNREE  80

NP_001165980.1   81   HAEFVLVRKDVLFSQLVETALLALGYSHSSAAQAQGIIKLGRWNPLPLSYVTDAPDATVADMLQDVYHVVTLKIQLQSCS  160
NP_631885.1      81   HAEFVLVRKDVLFSQLVETALLALGYSHSSAAQAQGIIKLGRWNPLPLSYVTDAPDATVADMLQDVYHVVTLKIQLQSCS  160
XP_005640560.1   81   HAEFVLVRKDVLFSQLVETALLALGYSHSSAAQAQGIIKLGRWNPLPLSYVTDAPDATVADMLQDVYHVVTLKIQLQSCS  160

NP_001165980.1   161  KLEDLPAEQWNHATVRNALKELLKEMNQSTLAKECPLSQSMISSIVNSTYYANVSATKCQEFGRWYKKYKKIKVERVERE  240
NP_631885.1      161  KLEDLPAEQWNHATVRNALKELLKEMNQSTLAKECPLSQSMISSIVNSTYYANVSATKCQEFGRWYKKYKKIKVERVERE  240
XP_005640560.1   161  KLEDLPAEQWNHATVRNALKELLKEMNQSTLAKECPLSQSMISSIVNSTYYANVSATKCQEFGRWYKKYKKIKVERVERE  240

NP_001165980.1   241  NLSDYCVLGQRPMHLPNMNQLASLGKTNEQSPHSQIHHSTPIRNQVPALQPIMSPGLLSPQLSPQLVRQQIAMAHLINQQ  320
NP_631885.1      241  NLSDYCVLGQRPMHLPNMNQLASLGKTNEQSPHSQIHHSTPIRNQVPALQPIMSPGLLSPQLSPQLVRQQIAMAHLINQQ  320
XP_005640560.1   241  NLSDYCVLGQRPMHLPNMNQLASLGKTNEQSPHSQIHHSTPIRNQVPALQPIMSPGLLSPQLSPQLVRQQIAMAHLINQQ  320

NP_001165980.1   321  IAVSRLLAHQHPQAINQQFLNHPPIPRAVKPEPTNSSVEVSPDIYQQVRDELKRASVSQAVFARVAFNRTQGLLSEILRK  400
NP_631885.1      321  IAVSRLLAHQHPQAINQQFLNHPPIPRAVKPEPTNSSVEVSPDIYQQVRDELKRASVSQAVFARVAFNRTQGLLSEILRK  400
XP_005640560.1   321  IAVSRLLAHQHPQAINQQFLNHPPIPRAVKPEPTNSSVEVSPDIYQQVRDELKRASVSQAVFARVAFNRTQGLLSEILRK  400

NP_001165980.1   401  EEDPRTASQSLLVNLRAMQNFLNLPEVERDRIYQDERERSMNPNVSMVSSASSSPSSSRTPQAKTSTPTTDLPIKVDGAN  480
NP_631885.1      401  EEDPRTASQSLLVNLRAMQNFLNLPEVERDRIYQDERERSMNPNVSMVSSASSSPSSSRTPQAKTSTPTTDLPIKVDGAN  480
XP_005640560.1   401  EEDPRTASQSLLVNLRAMQNFLNLPEVERDRIYQDERERSMNPNVSMVSSASSSPSSSRTPQAKTSTPTTDLPIKVDGAN  480

NP_001165980.1   481  INITAAIYDEIQQEMKRAKVSQALFAKVAANKSQGWLCELLRWKENPSPENRTLWENLCTIRRFLNLPQHERDVIYEEES  560
NP_631885.1      481  VNITAAIYDEIQQEMKRAKVSQALFAKVAANKSQGWLCELLRWKENPSPENRTLWENLCTIRRFLNLPQHERDVIYEEES  560
XP_005640560.1   481  VNITAAIYDEIQQEMKRAKVSQALFAKVAANKSQGWLCELLRWKENPSPENRTLWENLCTIRRFLNLPQHERDVIYEEES  560

NP_001165980.1   561  RHHHSERMQHVVQLPPEPVQVLHRQQSQPAKESSPPREEAPPPPPPTEDSCAKKPRSRTKISLEALGILQSFIHDVGLYP  640
NP_631885.1      561  RHHHSERMQHVVQLPPEPVQVLHRQQSQPTKESSPPREEAPPPPPPTEDSCAKKPRSRTKISLEALGILQSFIHDVGLYP  640
XP_005640560.1   561  RHHHSERMQHVVQLPPEPVQVLHRQQSQPAKESSPPREEAPPPPPPTEDSCAKKPRSRTKISLEALGILQSFIHDVGLYP  640

NP_001165980.1   641  DQEAIHTLSAQLDLPKHTIIKFFQNQRYHVKHHGKLKEHLGSAVDVAEYKDEELLTESEENDSEEGSEEMYKVEAEEENA  720
NP_631885.1      641  DQEAIHTLSAQLDLPKHTIIKFFQNQRYHVKHHGKLKEHLGSAVDVAEYKDEELLTESEENDSEEGSEEMYKVEAEEENA  720
XP_005640560.1   641  DQEAIHTLSAQLDLPKHTIIKFFQNQRYHVKHHGKLKEHLGSAVDVAEYKDEELLTESEENDSEEGSEEMYKVEAEEENA  720

NP_001165980.1   721  DKSKAAPAEIDQR  733
NP_631885.1      721  DKSKAAPAETDQR  733
XP_005640560.1   721  EKSKATPAEIDQR  733
```

CD31

```
NP_000433.4      1    MQPRWAQGATMWLGVLLTLLLCSSLEGQENSFTINSVDMKSLPDWTVQNGKNLTLQCFADVSTTSHVKPQHQMLFYKDDV  80
NP_001292086.1   1    ----------MLLALGLTLVLYASLQAEENSFTINSIHMESLPSWEVMNGQQLTLECLVDISTTSKSRSQHRVLFYKDDA  70
XP_022278549.1   1    MQLEWTQEGKMWLGALLILLLCPSLEGQENSFTINDIHMEILPGEEVQNGENMTLQCIVDISTTSHIKPQHWVLFYKDDV  80

NP_000433.4      81   LFYNISSMKSTESYFIPEVRIYDSGTYKCTVIVNNKEKTTAEYQVLVEGVPSPRVTLDKKEAIQGGIVRVNCSVPEEKAP  160
NP_001292086.1   71   MVYNVTSREHTESYVIPQARVFHSGKYKCTVMLNNKEKTTIEYEVKVHGVSKPKVTLDKKEVTEGGVVTVNCSLQEEKPP  150
XP_022278549.1   81   LFHNVSSVENTESYFIPRARVYDAGTYKCTVILNNKEKTSLEYQVWVKGVSDPIVTLDKKEAIEGGVVKVNCSVPEEKPP  160

NP_000433.4      161  IHFTIEKLELNEKMVKLKREKNSRDQNFVILEFPVEEQDRVLSFRCQARIISGIHMQTSESTKSELVTVTESFSTPKFHI  240
NP_001292086.1   151  IFFKIEKLEVGTKFVKRRIDKTS-NENFVLMEFPIEAQDHVLVFRCQAGILSGFKLQESEPIRSEYVTVQESFSTPKFEI  229
XP_022278549.1   161  IHFTIEKLKLDTKGFKQKREKTSPNRNFMMLEFTVEEQDHVIFFQCQARIISGTHMETSRAIKSELVTVTESFSNPKFHV  240

NP_000433.4      241  SPTGMIMEGAQLHIKCTIQVTHLAQEFPEIIIQKDKAIVAHNRHGNKAVYSVMAMVEHSGNYTCKVESSRISKVSSIVVN  320
NP_001292086.1   230  KPPGMIIEGDQLHIRCIVQVTHLVQEFTEIIIQKDKAIVATSKQSSEAVYSVMAMVEYSGHYTCKVESNRISKASSIMVN  309
XP_022278549.1   241  SPEGVITEGDQLYIRCTIQVTHLVQAFPEIIIQKDKAIVAHKRHGNEATYSVMAMAEHNGNYTCKVEASRISKVSSIVVN  320

NP_000433.4      321  ITELFSKPELESSFTHLDQGERLNLSCSIPGAPPANFTIQKEDTIVSQTQDFTKIASKSDSGTYICTAGIDKVVKKSNTV  400
NP_001292086.1   310  ITELFPKPKLEFSSSRLDQGELLDLSCSVSGTPVANFTIQKEETVLSQYQNFSKIAEESDSGEYSCTAGIGKVVKRSGLV  389
XP_022278549.1   321  ITELFSKPKLESSITRLDQGESLNLWCSIPEAPPANFTIQKENTIVSQSQNFTKIASATDSGTYTCNASMGKVVKRSSAV  400

NP_000433.4      401  QIVVCEMLSQPRISYDAQFEVIKGQTIEVRCESISGTLPISYQLLKTSKVLENSTKNSNDPAVFKDNPTEDVEYQCVADN  480
NP_001292086.1   390  PIQVCEMLSKPSIFHDAKSEIIKGHAIGISCQSENGTAPITYHLMKAKSDFQTLEVTSNDPATFTDKPTRDMEYQCRADN  469
XP_022278549.1   401  QITVCEMLSKPRIFYDSSSEVIKGQTIAVSCQSINGTTPISYHLLKTSNILESRDMSSNEPAVFKDNPTKDTEYQCIVDN  480

NP_000433.4      481  CHSHAKMLSEVLRVKVIAPVDEVQISILSSKVVESGEDIVLQCAVNEGSGPITYKFYREKEGKPFYQMTSNATQAFWTKQ  560
NP_001292086.1   470  CHSHPAVFSEILRVRVIAPVDEVVISILSSNEVQSGSEMVLRCSVKEGTSPITFQFYKEKEDRPFHQAVVNDTQAFWHNK  549
XP_022278549.1   481  CHSHSEMVSEVLRVKVIAPVDEVKLSILMNAEVEFGKDIELLCSVNEATGPITYRFYKEA-GSLLYQITSNETHAVWYKS  559

NP_000433.4      561  KASKEQEGEYYCTAFNRANHASSVPRSKILTVRVILAPWKKGLIAVVIIGVIIALLIIAAKCYFLRKAKAKQMPVEMSRP  640
NP_001292086.1   550  QASKKQEGQYYCTASNRASSMRTSPRSSTLAVRVFLAPWKKGLIAVVVIGVVIATLIVAAKCYFLRKAKAKQKPVEMSRP  629
XP_022278549.1   560  KASKEDEGQYYCTASNRANRLKSSPQSNVLTVRVFLAAWIKGLIAVVVIGVIIGVLILGARCYILKKAKAKQTPVEMSRP  639

NP_000433.4      641  AVPLLNSNNEKM-SDPNMEANSHYGHNDDVRNHAMKPINDNKEPLNSDVQYTEVQVSSAESHKDLGKKDTETVYSEVRKA  719
NP_001292086.1   630  AAPLLNSNSEKI-SEPSVEANSHYGYDDVSGNDAVKPINQNKDPQNMDVEYTEVEVSSLEPHQALGTRATETVYSEIRKV  708
XP_022278549.1   640  AVPLLNSTNEKMlSDPNTEVNRHYGYNEDVGNHAMKPINENKEPLTLDVEYTEVEVTSPEPYQGLETKGTETVYSEIRKA  719

NP_000433.4      720  VPDAVESRYSRTEGSLDGT   738
NP_001292086.1   709  DPNLMENRYSRTEGSLNGT   727
XP_022278549.1   720  NPDFMENRYSRTEGSLDGT   738
```

PROX1

```
NP_001257545.1   1    MPDHDSTALLSRQTKRRRVDIGVKRTVGTASAFFAKARATFFSAMNPQGSEQDVEYSVVQHADGEKSNVLRKLLKRANSY  80
NP_032963.1      1    MPDHDSTALLSRQTKRRRVDIGVKRTVGTASAFFAKARATFFSAMNPQGSEQDVEYSVVQHADGEKSNVLRKLLKRANSY  80
XP_005622418.1   1    MPDHDSTALLSRQTKRRRVDIGVKRTVGTASAFFAKARATFFSAMNPQGSEQDVEYSVVQHADGEKSNVLRKLLKRANSY  80

NP_001257545.1   81   EDAMMPFPGATIISQLLKNNMNKNGGTEPSFQASGLSSTGSEVHQEDICSNSSRDSPPECLSPFGRPTMSQFDMDRLCDE  160
NP_032963.1      81   EDAMMPFPGATIISQLLKNNMNKNGGTEPSFQASGLSSTGSEVHQEDICSNSSRDSPPECLSPFGRPTMSQFDVDRLCDE  160
XP_005622418.1   81   EDAMMPFPGATIISQLLKNNMNKNGGTEPSFQASGLSSTGSEVHQEDICSNSSRDSPPECLSPFGRPTMSQFDMDRLCDE  160

NP_001257545.1   161  HLRAKRARVENIIRGMSHSPSVALRGNENEREMAPQSVSPRESYRENKRKQKLPQQQQQSFQQLVSARKEQKREERRQLK  240
NP_032963.1      161  HLRAKRARVENIIRGMSHSPSVALRGNENEREMAPQSVSPRESYRENKRKQKLPQQQQQSFQQLVSARKEQKREERRQLK  240
XP_005622418.1   161  HLRAKRARVENIIRGMSHSPSVALRGNENEREMAPQSVSPRESYRENKRKQKLPQQQQQSFQQLVSARKEQKREERRQLK  240

NP_001257545.1   241  QQLEDMQKQLRQLQEKFYQIYDSTDSENDEDGNLSEDSMRSEILDARAQDSVGRSDNEMCELDPGQFIDRARALIREQEM  320
NP_032963.1      241  QQLEDMQKQLRQLQEKFYQVYDSTDSENDEDGDLSEDSMRSEILDARAQDSVGRSDNEMCELDPGQFIDRARALIREQEM  320
XP_005622418.1   241  QQLEDMQKQLRQLQEKFYQIYDSTDSENDEDGNLSEDSMRSEILDARAQDSVGRSDNEMCELDPGQFIDRARALIREQEL  320

NP_001257545.1   321  AENKPKREGNNKERDHGPNSLQPEGKHLAETLKQELNTAMSQVVDTVVKVFSAKPSRQVPQVFPPLQIPQARFAVNGENH  400
NP_032963.1      321  AENKPKREGSNKERDHGPNSLQPEGKHLAETLKQELNTAMSQVVDTVVKVFSAKPSRQVPQVFPPLQIPQARFAVNGENH  400
XP_005622418.1   321  AENKPKREGNNKERDHGPNSLQPEGKHLAETLKQELNTAMSQVVDTVVKVFSAKPSRQVPQVFPPLQIPQARFAVNGENH  400

NP_001257545.1   401  NFHTANQRLQCFGDVIIPNPLDTFGNVQMASSTDQTEALPLVVRKNSSDQSASGPAAGGHHQPLHQSPLSATTGFTTSTF  480
NP_032963.1      401  NFHTANQRLQCFGDVIIPNPLDTFGSVQMPSSTDQTEALPLVVRKNSSEQSASGPATGGHHQPLHQSPLSATAGFTTPSF  480
XP_005622418.1   401  NFHTANQRLQCFGDVIIPNPLDTFGNVQMPSSTDQTEALPLVVRKNSSDQSASGPPAGGHHQPLHQSPLSATAGFTTSTF  480

NP_001257545.1   481  RHPFPLPLMAYPFQSPLGAPSGSFSGKDRASPESLDLTRDTTSLRTKMSSHHLSHHPCSPAHPPSTAEGLSLSLIKSECG  560
NP_032963.1      481  RHPFPLPLMAYPFQSPLGAPSGSFSGKDRASPESLDLTRDTTSLRTKMSSHHLSHHPCSPAHPPSTAEGLSLSLIKSECG  560
```

XP_005622418.1 481 RHPFPLPLMAYPFQSPLGAPSGSFSGKDRASPESLDLTRETTSLRTKMSSHHLSHHPCSPAHPPSTAEGLSLSLIKSECG 560

NP_001257545.1 561 DLQDMSEISPYSGSAMQEGLSPNHLKKAKLMFFYTRYPSSNMLKTYFSDVKFNRCITSQLIKWFSNFREFYYIQMEKYAR 640
NP_032963.1 561 DLQDMSDISPYSGSAMQEGLSPNHLKKAKLMFFYTRYPSSNMLKTYFSDVKFNRCITSQLIKWFSNFREFYYIQMEKYAR 640
XP_005622418.1 561 DLQDMSEISPYSGSAMQEGLSPNHLKKAKLMFFYTRYPSSNMLKTYFSDVKFNRCITSQLIKWFSNFREFYYIQMEKYAR 640

NP_001257545.1 641 QAINDGVTSTEELSITRDCELYRALNMHYNKANDFE-VPERFLEVAQITLREFFNAIIAGKDVDPSWKKAIYKVICKLDS 719
NP_032963.1 641 QAINDGVTSTEELSITRDCELYRALNMHYNKANDFE-VPERFLEVAQITLREFFNAIIAGKDVDPSWKKAIYKVICKLDS 719
XP_005622418.1 641 QAINDGVTSTEELSITRDCELYRALNMHYNKANDFEqVPERFLEVAQITLREFFNAIIAGKDVDPSWKKAIYKVICKLDS 720

NP_001257545.1 720 EVPEIFKSPNCLQELLHE 737
NP_032963.1 720 EVPEIFKSPNCLQELLHE 737
XP_005622418.1 721 EVPEIFKSPNCLQELLHE 738

Vimentin

NP_003371.2 1 MSTRSVSSSSYRRMFGGPGTASRPSSSRSYVTTSTRTYSLGSALRPSTSRSLYASSPGGVYATRSSAVRLRSSVPGVRLL 80
NP_035831.2 1 MSTRSVSSSSYRRMFGGSGTSSRPSSNRSYVTTSTRTYSLGSALRPSTSRSLYSSSPGGAYVTRSSAVRLRSSVPGVRLL 80
NP_001273952.1 1 MSTRSVSSSSYRRMFGGPGTGSRPSSTRSYVTTSTRTYSLGSALRPSTSRSLYASSPGGAYATRSSAVRLRSSVPGVRLL 80

NP_003371.2 81 QDSVDFSLADAINTEFKNTRTNEKVELQELNDRFANYIDKVRFLEQQNKILLAELEQLKGQGKSRLGDLYEEEMRELRRQ 160
NP_035831.2 81 QDSVDFSLADAINTEFKNTRTNEKVELQELNDRFANYIDKVRFLEQQNKILLAELEQLKGQGKSRLGDLYEEEMRELRRQ 160
NP_001273952.1 81 QDSVDFSLADAINTEFKNTRTNEKVELQELNDRFANYIDKVRFLEQQNKILLAELEQLKGQGKSRLGDLYEEEMRELRRQ 160

NP_003371.2 161 VDQLTNDKARVEVERDNLAEDIMRLREKLQEEMLQREEAENTLQSFRQDVDNASLARLDLERKVESLQEEIAFLKKLHEE 240
NP_035831.2 161 VDQLTNDKARVEVERDNLAEDIMRLREKLQEEMLQREEAESTLQSFRQDVDNASLARLDLERKVESLQEEIAFLKKLHDE 240
NP_001273952.1 161 VDQLTNDKARVEVERDNLAEDIMRLREKLQEEMLQREEAESTLQSFRQDVDNASLARLDLERKVESLQEEIAFLKKLHDE 240

NP_003371.2 241 EIQELQAQIQEQHVQIDVDVSKPDLTAALRDVRQQYESVAAKNLQEAEEWYKSKFADLSEAANRNNDALRQAKQESTEYR 320
NP_035831.2 241 EIQELQAQIQEQHVQIDVDVSKPDLTAALRDVRQQYESVAAKNLQEAEEWYKSKFADLSEAANRNNDALRQAKQESNEYR 320
NP_001273952.1 241 EIQELQAQIQDQHVQIDMDVSKPDLTAALRDVRQQYESVAAKNLQEAEEWYKSKFADLSEAANRNNDALRQAKQESNEYR 320

NP_003371.2 321 RQVQSLTCEVDALKGTNESLERQMREMEENFAVEAANYQDTIGRLQDEIQNMKEEMARHLREYQDLLNVKMALDIEIATY 400
NP_035831.2 321 RQVQSLTCEVDALKGTNESLERQMREMEENFALEAANYQDTIGRLQDEIQNMKEEMARHLREYQDLLNVKMALDIEIATY 400
NP_001273952.1 321 RQVQSLTCEVDALKGTNESLERQMREMEENFAVEAANYQDTIGRLQDEIQNMKEEMARHLREYQDLLNVKMALDIEIATY 400

NP_003371.2 401 RKLLEGEESRISLPLPNFSSLNLRETNLDSLPLVDTHSKRTLLIKTVETRDGQVINETSQHHDDLE 466
NP_035831.2 401 RKLLEGEESRISLPLPTFSSLNLRETNLESLPLVDTHSKRTLLIKTVETRDGQVINETSQHHDDLE 466
NP_001273952.1 401 RKLLEGEESRIALPLPNFSSLNLRETNLDSLPLVDTHSKRTLLIKTVETRDGQVINETSQHHDDLE 466

FAPa

NP_004451.2 1 MKTWVKIVFGVATSAVLALLVMCIVLRPSRVHNSEENTMRALTLKDILNGTFSYKTFFPNWISGQEYLHQSADNNIVLYN 80
NP_032012.1 1 MKTWLKTVFGVTTLAALALVVICIVLRPSRVYKPEGNTKRALTLKDILNGTFSYKTYFPNWISEQEYLHQSEDDNIVFYN 80
XP_005640309.2 1 MKTWLKIVFGVATSAVLALLVMCIVLRPSRVHDSEGGTTRALTLEDILNGTFTYKTFFPNWISGQEYLHQSTDNDIVYYN 80

NP_004451.2 81 IETGQSYTILSNRTMKSVNASNYGLSPDRQFVYLESDYSKLWRYSYTATYYIYDLSNGEFVRGNELPRPIQYLCWSPVGS 160
NP_032012.1 81 IETRESYIILSNSTMKSVNATDYGLSPDRQFVYLESDYSKLWRYSYTATYYIYDLQNGEFVRGYELPRPIQYLCWSPVGS 160
XP_005640309.2 81 IETGESYTILSNATMKSVNASNYGLSPDRQFAYLESDYSKLWRYSYTATYHIYNLNNGEFIRRNELPRPIQYLCWSPVGS 160

NP_004451.2 161 KLAYVYQNNIYLKQRPGDPPFQITFNGRENKIFNGIPDWVYEEEMLATKYALWWSPNGKFLAYAEFNDTDIPVIAYSYYG 240
NP_032012.1 161 KLAYVYQNNIYLKQRPGDPPFQITYTGRENRIFNGIPDWVYEEEMLATKYALWWSPDGKFLAYVEFNDSDIPIIAYSYYG 240
XP_005640309.2 161 KLAYVYQNNIYLKQRPEDPPFQITYNGRENKIFNGIPDWVYEEEMLATKHALWWSPNGKFLAYAEFNDTEIPVIAYSYYG 240

NP_004451.2 241 DEQYPRTINIPYPKAGAKNPVVRIFIIDTTYPAYVGPQEVPVPAMIASSDYYFSWLTWVTDERVCLQWLKRVQNVSVLSI 320
NP_032012.1 241 DGQYPRTINIPYPKAGAKNPVVRVFIVDTTYPHHVGPMEVPVPEMIASSDYYFSWLTWVSSERVCLQWLKRVQNVSVLSI 320
XP_005640309.2 241 DEQYPRTINIPYPKAGAKNPVVRIFIIDTTYPQQTGPREVPVPAMIASSDYYFSWLTWVTDERVCLQWLKRIQNVSVLSI 320

NP_004451.2 321 CDFREDWQTWDCPKTQEHIEESRTGWAGGFFVSTPVFSYDAISYYKIFSDKDGYKHIHYIKDTVENAIQITSGKWEAINI 400
NP_032012.1 321 CDFREDWHAWECPKNQEHVEESRTGWAGGFFVSTPAFSQDATSYYKIFSDKDGYKHIHYIKDTVENAIQITSGKWEAIYI 400
XP_005640309.2 321 CDFREGWQTWDCPKAQEHIEESRTGWAGGFFVSTPVFSYDAISYYKIFSDKDGYKHIHYIKDTVENAIQITSGKWEAINI 400

NP_004451.2 401 FRVTQDSLFYSSNEFEEYPGRRNIYRISIGSYPPSKKCVTCHLRKERCQYYTASFSDYAKYYALVCYGPGIPISTLHDGR 480
NP_032012.1 401 FRVTQDSLFYSSNEFEGYPGRRNIYRISIGNSPPSKKCVTCHLRKERCQYYTASFSYKAKYYALVCYGPGLPISTLHDGR 480
XP_005640309.2 401 FRVTQDSLFYSSNEFEDYPGRRNIYRISIGSSPPSKKCITCHLRKERCQYYTASFSDYAKYYALICYGPGLPISTLHDGH 480

NP_004451.2 481 TDQEIKILEENKELENALKNIQLPKEEIKKLEVDEITLWYKMILPPQFDRSKKYPLLIQVYGGPCSQSVRSVFAVNWISY 560

```
NP_032012.1      481  TDQEIQVLEENKELENSLRNIQLPKVEIKKLKDGGLTFWYKMILPPQFDRSKKYPLLIQVYGGPCSQSVKSVFAVNWITY  560
XP_005640309.2   481  TDQEIKILEENKELENALKNIQLPKEEIKKLEVDDITLWYKMMLPPRFDRSKKYPLLIQVYGGPCSQSVKSVFSINWISY  560

NP_004451.2      561  LASKEGMVIALVDGRGTAFQGDKLLYAVYRKLGVYEVEDQITAVRKFIEMGFIDEKRIAIWGWSYGGYVSSLALASGTGL  640
NP_032012.1      561  LASKEGIVIALVDGRGTAFQGDKFLHAVYRKLGVYEVEDQLTAVRKFIEMGFIDEERIAIWGWSYGGYVSSLALASGTGL  640
XP_005640309.2   561  LASKEGIVIALVDGRGTAYQGDKLLYAVYRKLGVYEVEDQITAVRKFIEMGFIDEKRIAIWGWSYGGYVSSLALASGTGL  640

NP_004451.2      641  FKCGIAVAPVSSWEYYASVYTERFMGLPTKDDNLEHYKNSTVMARAEYFRNVDYLLIHGTADDNVHFQNSAQIAKALVNA  720
NP_032012.1      641  FKCGIAVAPVSSWEYYASIYSERFMGLPTKDDNLEHYKNSTVMARAEYFRNVDYLLIHGTADDNVHFQNSAQIAKALVNA  720
XP_005640309.2   641  FKCGIAVAPVSSWEYYASIYTERFMGLPTKNDNLEHYKNSTVMARAEYFRNVDYLLIHGTADDNVHFQNSAQIAKALVNA  720

NP_004451.2      721  QVDFQAMWYSDQNHGL-SGLSTNHLYTHMTHFLKQCFSLSD  760
NP_032012.1      721  QVDFQAMWYSDQNHGIsSGRSQNHLYTHMTHFLKQCFSLSD  761
XP_005640309.2   721  QVDFQAMWYSDQNHGI-PGLSSKHLYTRMTHFLKQCFSLSD  760
```

CD20

```
NP_061883.1  1    MDFSRRSFHRSLSSSLQAPVVSTVG-------MQRLGTTPSVYGGAGGRGIRISNSRHTVNYGSDLTGGGDLFVGNEKMA  73
NP_075745.1  1    MDFSRQSFHRSLSSSSQGPALSMSGSLYRKGTVQRLGAAPSVYGGAGGHGTRISVSKAVMSYGGDLSNGSDLFGGNGKLA  80
XP_850091.2  1    MEFSRRNFHRGLSSSPQTSALSMSGSMYRKEDMQYFGAAPSVYGGAGGQGIRISTSRHMRGYGGDHTKG-NLFVGNEKTT  79

NP_061883.1  74   MQNLNDRLASYLEKVRTLEQSNSKLEVQIKQWYETNAPRAGRDYSAYYRQIEELRSQIKDAQLQNARCVLQIDNAKLAAE  153
NP_075745.1  81   MQNLNDRLANYLEKVRSLEQSNSRLEAQIKQWYETNAPSTIRDYSSYYAQIKELQNQVKDAQVQNAQCVLRIDNAKLAAE  160
XP_850091.2  80   MQNLNDRLASYLAKVQSLEKSNSKLESLIKEWYKTNTPSA-KDYSAYYKQMEELQKQIKKAQLENARCVLQIDNAKLAAE  158

NP_061883.1  154  DFRLKYETERGIRLTVEADLQGLNKVFDDLTLHKTDLEIQIEELNKDLALLKKEHQEEVDGLHKHLGNTVNVEVDAAPGL  233
NP_075745.1  161  DFRLKFETERGMRIAVEADLQGLSKVYDNLTLQKTDLEIQIEELNKDLALLKKEHQEEVEVLRRQLGNNVNVEVDAAPGL  240
XP_850091.2  159  DFRLRYETERGIRLTVESDLQGLNKVLDDLALTRKDLEIQVKELSKDLDILKKEHQEEVDSLHRHLGNTVSVELDAAPAL  238

NP_061883.1  234  NLGVIMNEMRQKYEVMAQKNLQEAKEQFERQTAVLQQQVTVNTEELKGTEVQLTELRRTSQSLEIELQSHLSMKESLEHT  313
NP_075745.1  241  NLGEIMNEMRQRYEVLAQKNLQEAKEQFERQSQTLQQQVTVNTEELKGFEVQVTELRRTYQNLEIELQSHLSMKESLERN  320
XP_850091.2  239  NLGTTMNEMRQKYEVIAQENLQKAKEQFEKQTESLQQQVIGSNEELKEAEFQVKELRRTYQNLEIELQSLLSLKETLEHT  318

NP_061883.1  314  LEETKARYSSQLANLQSLLSSLEAQLMQIRSNMERQNNEYHILLDIKTRLEQEIATYRRLLEGED   VKTTEYQLSTLE  390
NP_075745.1  321  LEDVKARYASQLAAIQEMLSSLEAQLMQIRSDTERQNQEHNILLDIKTRLEQEIATYRRLLEGED   IKTTEYQLSTLE  397
XP_850091.2  319  LDNTKDCYSHKLATIQTVLDNLEVQLTQIRAETEYQSNEYNILFDIKTRLEQEIATYRRLLEGED[5]ITIPDDQIHIEE  400

NP_061883.1  391  ERDIKKTRKIKTVVQEVVDGKVVSSEVKEVEENI  424
NP_075745.1  398  MKDIKKTRKIKTVVEEVVDGKVVSSEVKEIEESV  431
XP_850091.2  401  EKDIKRTRKIKTVVEEVVNGMVVSSETREVEENM  434
```

Iba1

```
NP_001614.3  1    MSQTRDLQGGKAFGLLKAQQEERLDEINKQFLDDPKYSSDEDLPSKLEGFKEKYMEFDLNGNGDIDIMSLKRMLEKLGVP  80
NP_062340.1  1    MSQSRDLQGGKAFGLLKAQQEERLEGINKQFLDDPKYSNDEDLPSKLEAFKVKYMEFDLNGNGDIDIMSLKRMLEKLGVP  80
XP_532072.2  1    MSQTRDLQGGKAFGLLKAQQEDRLDEINKQFLDDPKYSSDEDLPSKLEAFKKKYMEFDLNGDGDIDIMALKRMLEKLGVP  80

NP_001614.3  81   KTHLELKKLIGEVSSGSGETFSYPDFLRMMLGKRSAILKMILMYEEKAREKEKPTGPPAKKAISELP  147
NP_062340.1  81   KTHLELKRLIREVSSGSEETFSYSDFLRMMLGKRSAILRMILMYEEKNKEHKRPTGPPAKKAISELP  147
XP_532072.2  81   KTHLELKKLIREVSSSSGETFSYSDFLKMMLGKKSAILKIILMYEEKAREQEKPAGPPAKKDISELP  147
```

FOXP3

```
AAI43787.1       1    MPNPRPGKPSAPSLALGPSPGASPSWRAAPKASDLLGARGPGGTFQGRDLRGGAHASSSsLNPMPPSQLQLPTLPLVMVA  80
AAI32336.1       1    MPNPRPAKPMAPSLALGPSPGVLPSWKTAPKGSELLGTRGSGGPFQGRDLRSGAHTSSS-LNPLPPSQLQLPTVPLVMVA  79
NP_001161933.1   1    MPNPRPAKPSAPSLAPGPSPGALPSWRAAPKASDLLGAKGPGVTFQGRDLRGGTHASSS-LNPMPPSQLQLPTVPLVMVA  79

AAI43787.1       81   PSGARLGPLPHLQALLQDRPHFMHQLSTVDAHARTPVLQVHPLESPAMISLTPPTTATGVFSLKARPGLPPGINVASLEW  160
AAI32336.1       80   PSGARLGPSPHLQALLQDRPHFMHQLSTVDAHAQTPVLQVRPLDNPAMISLPPPSAATGVFSLKARPGLPPGINVASLEW  159
NP_001161933.1   80   PSGARLGPSPHLQALLQDRPHFMHQLSTVGTHTRTPVLQVRPLDSPAMISLPPPTAATSVFSLKARPGLPPGINVASLEW  159

AAI43787.1       161  VSREPALLCTFPNPSAPRKD[23]STLSAVPQSSYPLLANGVCKWPGCEKVFEEPEDFLKHCQADHLLDEKGRAQCLLQR  259
AAI32336.1       160  VSREPALLCTFPRSGTPRKD    SNLLAAPQGSYPLLANGVCKWPGCEKVFEEPEEFLKHCQADHLLDEKGKAQCLLQR  235
NP_001161933.1   160  VSREPALLCTFPSPSTPRKD    STLPTVPQGSYSLLANGVCKWPGCEKVFEEPEDFLKHCQADHLLDEKGRAQCLLQR  235
```

```
AAI43787.1       260  EMVQSLEQQLVLEKEKLSAMQAHLAGKMALTKASSVASSDKGSCCIVAAGSQGPVVPAWSGPREAPD-SLFAVRRHLWGS  338
AAI32336.1       236  EVVQSLEQQLELEKEKLGAMQAHLAGKMALAKAPSVASMDKSSCCIVATSTQGSVLPAWSAPREAPDgGLFAVRRHLWGS  315
NP_001161933.1   236  EVVQSLEQQLVLEKEKLGAMQAHLAGKMTLTKAPSTASSDKGSCCIVAAGTPATTGPAWSSPQEAPD-GLFAVRRHLWGS  314

AAI43787.1       339  HGNSTFPEFLHNMDYFKFHNMRPPFTYATLIRWAILEAPEKQRTLNEIYHWFTRMFAFFRNHPATWKNAIRHNLSLHKCF  418
AAI32336.1       316  HGNSSFPEFFHNMDYFKYHNMRPPFTYATLIRWAILEAPERQRTLNEIYHWFTRMFAYFRNHPATWKNAIRHNLSLHKCF  395
NP_001161933.1   315  HGNSTFPEFFHNMDYFKFHNMRPPFTYATLIRWAILEAPEKQRTLNEIYHWFTRMFAFFRNHPATWKNAIRHNLSLHKCF  394

AAI43787.1       419  VRVESEKGAVWTVDELEFRKKRSQRPSRCSNPTPGP  454
AAI32336.1       396  VRVESEKGAVWTVDEFEFRKKRSQRPNKCSNPCP--  429
NP_001161933.1   395  VRVESEKGAVWTVDEFEFRKKRSQRPSRSSNPTPGP  430
```

CD3

```
NP_000724.1      1    MQSGTHWRVLGLCLLSVGVWGQD   GNEEMGGIT-QTPYKVSISGTTVILTCPQYPGSE-ILWQHNDKNIGGDEDD[8]  80
NP_031674.1      1    MRWNTFWGILCLSLLAVGTCQDD   --------AENIEYKVSISGTSVELTCP-LDSDENLKWEKNGQELPQKHDK     65
XP_038364988.1   1    MQSRNLWRILGLCLLSVGAWGQD[4]ASDDLTSISPEKRFKVSISGTEVVVTCPDVFGYDNIKWEKNDNLVEGASNR     78

NP_000724.1      81   HLSLKEFSELEQSGYYVCYPRGSKPEDanfYLYLRARVCENCMEMDVMSVATIVIVDICITGGLLLLVYYWSKNRKAKAK  160
NP_031674.1      66   HLVLQDFSEVEDSGYYVCYTPASNKNT---YLYLKARVCEYCVEVDLTAVAIIIIVDICITLGLLMVIYYWSKNRKAKAK  142
XP_038364988.1   79   ELSQKEFSEVDDSGYYACYADSIKEKS---YLYLRARVCANCIEVNLMAVVTIIVADICLTLGLLMVYYWSKTRKANAK  155

NP_000724.1      161  PVTRGAGAGGRQRGQNKERPPPVPNPDYEPIRKGQRDLYSGLNQRRI  207
NP_031674.1      143  PVTRGTGAGSRPRGQNKERPPPVPNPDYEPIRKGQRDLYSGLNQRAV  189
XP_038364988.1   156  PVMRGTGAGSRPRGQNKEKPPPVPNPDYEPIRKGQQDLYSGLNQRGI  202
```
